# Ultradifferentiable CR Manifolds

**DOI:** 10.1007/s12220-019-00191-6

**Published:** 2019-04-18

**Authors:** Stefan Fürdös

**Affiliations:** 1 Faculty of Mathematics, University of Vienna, Oskar-Morgenstern-Platz 1, 1090 Vienna, Austria; 2 Present Address: Department of Mathematics and Statistics, Masaryk University, Kotlarska 2, 611 37 Brno, Czech Republic

**Keywords:** Ultradifferentiable CR manifolds, Ultradifferentiable regularity, CR mappings, Infinitesimal CR automorphisms, 32V05, 32V99, 26E10, 35A18

## Abstract

In this article, the notion of ultradifferentiable CR manifold is introduced and an ultradifferentiable regularity result for finitely nondegenerate CR mappings is proven. Here, ultradifferentiable means with respect to Denjoy–Carleman classes defined by weight sequences. Furthermore, the regularity of infinitesimal CR automorphisms on ultradifferentiable abstract CR manifolds is investigated.

## Introduction

The primary focus of this article is the study of the regularity of CR mappings. Looking at the literature concerning this problem, one observes that most theorems about the regularity of CR mappings are of a similar form which can be summarised as follows: We consider a CR mapping *H* between two CR submanifolds *M* and $M^\prime$ with some a priori regularity that extends to a holomorphic mapping defined on a wedge with edge *M*. If the mapping and/or the manifolds satisfy certain nondegeneracy conditions at some point then it is proven that *H* is actually of optimal regularity near this point, that is smooth if *M* and $M^\prime$ are smooth, or real analytic if the manifolds are real analytic. We should mention that the nondegeneracy assumptions are heavily tailored towards the methods applied in the various different proofs. In particular, it is worth noting that in most instances the conditions in the smooth setting differ sharply from those used in the analytic category. In the case of smooth CR manifolds, the fundamental contributions are the pioneering works of Fefferman [13] and Nirenberg–Webster–Yang [28]. We should also notice that in the analytic setting surprisingly weak assumptions often suffice, c.f. e.g. the classical results of Baouendi–Jacobowitz–Treves [28], Huang [20] and Pinčuk [30].

One of the rare cases, where under the identical assumptions it has been possible to show that *H* is smooth if the manifolds are smooth and analytic if *M* and $M^\prime$ are both analytic manifolds, has been the results of Lamel [23, 24]. He proved that every finitely nondegenerate CR mapping between two generic submanifolds that extends holomorphically is smooth and even analytic if both manifolds are real analytic.

Recently, Berhanu–Xiao [3] were able to strengthen this result in the smooth case by relaxing partially its assumptions. They require only the target manifold to be an embedded CR manifold, the source manifold could be only an abstract CR manifold. The finitely nondegenerate condition on the mapping remains unchanged but the holomorphic extension obviously makes no sense in this situation. It is replaced in the theorem of Berhanu–Xiao with the assumption that the fibres of the wavefront set of *H* do not include opposite directions.

This microlocal assumption is automatically satisfied in the embedded setting if extension to a wedge is assumed since Baouendi–Chang–Treves [1] showed that for CR distributions on CR submanifolds of ${\mathbb {C}}^N$, the holomorphic extension into wedges is in fact a microlocal condition, which they used to define the hypoanalytic wavefront set of CR distributions. It coincides with the analytic wavefront set if the manifold is analytic. If the manifold is only smooth then the hypoanalytic wavefront set includes the smooth wavefront set.

Since the results of Lamel and Berhanu–Xiao suggest that finite nondegeneracy preserves regularity quite well, the following question arises naturally. Given a subsheaf ${\mathcal {A}}$ of the sheaf of smooth functions, we may ask that if in the formulation of the theorem of Lamel the manifolds are assumed to be of class ${\mathcal {A}}$, does it follow that the CR mapping has to be of class ${\mathcal {A}}$ as well?

Of course, we have to assume that ${\mathcal {A}}$ satisfies certain properties. First of all, in order for the conjecture above to make sense, ${\mathcal {A}}$ must be closed under composition and the implicit function theorem must hold in the category of mappings of class ${\mathcal {A}}$. Furthermore, if we try to modify the existing proofs in the smooth category then we need some version of ${\mathcal {A}}$-wavefront set or more precisely a definition of ${\mathcal {A}}$-microlocal regularity. We should note at this point that in both Lamel’s proof and that of Berhanu–Xiao the characterisation of the smooth wavefront set by almost-analytic extensions was heavily used as both relied on an almost-analytic version of the implicit function theorem.

We are mainly interested in subsheafs of smooth functions that contain strictly the sheaf of real-analytic functions. We shall call the elements of such sheafs ultradifferentiable functions. Generally ultradifferentiable functions are determined either by estimates on its derivatives or its Fourier transform. The most well-known examples of ultradifferentiable classes are the Gevrey classes, see e.g. [32].

Here, we consider the category of the so-called Denjoy–Carleman classes, which are defined in the following way. If ${\mathcal {M}}=(m_j)_j$ is a sequence of positive real numbers then the Denjoy–Carleman class associated with ${\mathcal {M}}$ consists of those smooth functions that satisfy the following generalised Cauchy estimate1.1on compact sets, where *C* and *h* are constants independent of $\alpha$. We will also say that a smooth function *f* obeying (1.1) is of class $\{{\mathcal {M}}\}$. In particular, if ${\mathcal {M}}=(j!^s)_j$ then the associated Denjoy–Carleman class to ${\mathcal {M}}$ is the Gevrey class of order $s+1$.

Examining literature concerning the Denjoy–Carleman classes and their properties, one can observe that stability conditions of the associated class correlate with properties of the weight sequence. For example, we know that, if ${\mathcal {M}}$ is a regular weight sequence in the sense of [11], then the Denjoy–Carleman class associated with ${\mathcal {M}}$ is closed under composition, solving ordinary differential equations and the implicit function theorem holds in the class, c.f. e.g. [31]. Hence for regular sequences ${\mathcal {M}}$, we can consider manifolds of Denjoy–Carleman type. We shall say such a manifold is an ultradifferentiable manifold of class $\{{\mathcal {M}}\}$.

On the other hand, Hörmander [18] introduced the ultradifferentiable wavefront set for distributions defined on open subsets of the Euclidean space. But since he worked under comparatively weak conditions on the weight sequence, Hörmander was only able to define the ultradifferentiable wavefront set ${{\,\mathrm{WF}\,}}_{\mathcal {M}}u$ of distributions *u* on real-analytic manifolds but not distributions defined on ultradifferentiable manifolds.

However, using Dyn’kins characterisation of ultradifferentiable functions by almost-analytic extensions [10, 11], we were able in [14] to develop a geometric theory for the ultradifferentiable wavefront set. In particular, if the weight sequence is regular, the ultradifferentiable wavefront set of a distribution on an ultradifferentiable manifold is shown to be well defined. If we put additional conditions on the weight sequence, then we have also shown in [14] a microlocal elliptic regularity result for linear partial differential operators with ultradifferentiable coefficients acting on ultradifferentiable vector bundles. We are going to call weight sequences, that satisfy these conditions, normal.

With these results at hand and an ${\mathcal {M}}$-almost-analytic version of the almost-analytic implicit function theorem used in Lamel [24] and Berhanu–Xiao [3], it is possible to prove the ultradifferentiable version of the regularity result of Lamel:

### Theorem 1.1

Let ${\mathcal {M}}$ be a normal weight sequence and $M\subseteq {\mathbb {C}}^N$, $M^\prime \subseteq {\mathbb {C}}^{N^\prime }$ be two generic ultradifferentiable submanifolds of class $\{{\mathcal {M}}\}$, $p_0\in M$, $p^\prime _0\in M^\prime$ and $H:(M,p_0)\rightarrow (M^\prime ,p_0^\prime )$ a ${\mathcal {C}}^{k_0}$-CR mapping that is $k_0$-nondegenerate at $p_0$. Suppose furthermore that *H* extends continuously to a holomorphic map in a wedge ${\mathcal {W}}$ with edge *M*. Then *H* is ultradifferentiable of class $\{{\mathcal {M}}\}$ in a neighbourhood of $p_0$.

For the definition of finite nondegeneracy of a CR mapping, we refer to the beginning of Sect. 5 and for the definition of normal weight sequence to Sect. 2.

More precisely this paper is structured as follows. In Sect. 2, the necessary results on Denjoy–Carleman classes and ultradifferentiable manifolds are discussed. In Sect. 3, we first recall the results from Dyn’kin [10, 11] on the almost-analytic extension of ultradifferentiable functions. Furthermore, we give the definition of the ultradifferentiable wavefront set according to Hörmander [19] and close the section by briefly discussing the results on the ultradifferentiable wavefront set from [14], that are needed later on.

In Sect. 4, basic definitions and first results on ultradifferentiable CR manifolds are given, whereas the proofs of Theorem 1.1 and of ultradifferentiable versions of other regularity results of Lamel and Berhanu–Xiao are presented in Sect. 5. The last section is devoted to present essentially the generalisation of [15] concerning the smoothness of infinitesimal CR automorphisms to normal Denjoy–Carleman classes. We end by examining smooth infinitesimal CR automorphisms on formally holomorphic nondegenerate quasianalytic CR submanifolds.

We should note that although Theorem 1.1 gives rather precise information on the regularity of the mapping under consideration, the assumption of normality on the weight sequence is an obstruction, in the sense that any Denjoy–Carleman class given by a normal weight sequence is contained in some Gevrey class, c.f. [34]. It is possible to overcome this obstacle, but for that we have to work in a far more general setting, which will be done in a forthcoming paper.

## Denjoy–Carleman Classes

In this section, we summarise the results for Denjoy–Carleman classes that we need throughout the paper. For a more detailed presentation, see [14]. Note that, unless stated otherwise, $\Omega \subseteq {\mathbb {R}}^n$ will be an open set.

### Definition 2.1

Let ${\mathcal {M}}=(m_k)_k$ be a sequence of positive numbers. Then ${\mathcal {M}}$ is a regular weight sequence iff the following conditions hold.

M1 $$\begin{aligned} m_0=m_1=1 \end{aligned}$$

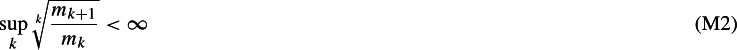

M3 $$\begin{aligned} m_k^2\le m_{k-1}m_{k+1}\qquad k\in {\mathbb {N}}\end{aligned}$$

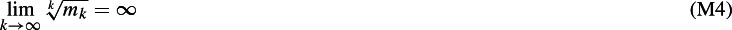


If ${\mathcal {M}}$ satisfies (M1), (M3), (M4) and

M2′ $$\begin{aligned} m_{j+k}\le Cq^{j+k}m_jm_k \qquad \forall (j,k)\in {\mathbb {N}}_0^2 \end{aligned}$$

for some constants $C,q>0$, then we say that ${\mathcal {M}}$ is a normal weight sequence.

Note that a normal weight sequence is regular, since (M2’) implies (M2), c.f. [21]. We shall also mention, that we assume that the weight sequence is normal only in those statements, whose proofs actually require the stronger condition on the weight sequence.

### Definition 2.2

Let ${\mathcal {M}}$ be a regular weight sequence. Then we say that a smooth function $f\in {\mathcal {E}}(\Omega )$ is ultradifferentiable of class $\{{\mathcal {M}}\}$ iff for all compact sets $K\subseteq \Omega$ there are constants $C,h>0$ such that

2.1 $$\begin{aligned} \bigl |\partial ^\alpha f(x)\bigr |\le Ch^{|\alpha |}m_{|\alpha |} |\alpha |! \end{aligned}$$

for all $x\in K$. The space of all ultradifferentiable functions of class $\{{\mathcal {M}}\}$ is denoted by ${\mathcal {E}}_{\mathcal {M}}(\Omega )$. It is sometimes also called the Denjoy–Carleman class associated with ${\mathcal {M}}$.

We say also that a mapping $F=(F_1,\cdots ,F_m):\,\Omega \rightarrow {\mathbb {R}}^m$ is of class $\{{\mathcal {M}}\}$, if all components $F_j$ are of class $\{{\mathcal {M}}\}$.

### Example 2.3

If $s> 0$ consider the normal weight sequence ${\mathcal {M}}^s=(k!^s)_k$. Its associated Denjoy–Carleman class is the Gevrey class ${\mathcal {G}}^{s+1}(\Omega )={\mathcal {E}}_{{\mathcal {M}}^s}(\Omega )$ of order $s+1$ on $\Omega$, c.f. [32].

On the other hand, the constant sequence ${\mathcal {M}}^0=(1)_k$ gives the space ${\mathcal {O}}(\Omega )$ of real-analytic functions on $\Omega$. Note that ${\mathcal {M}}^0$ is neither normal nor regular in the sense of Definition 2.1.

If ${\mathcal {M}}$ and ${\mathcal {N}}=(n_k)_k$ are two regular weight sequences then we write ${\mathcal {M}}\preccurlyeq {\mathcal {N}}$ iff there is a constant *Q* such that $m_k\le Q^{k+1}n_k$. It holds that ${\mathcal {E}}_{{\mathcal {M}}}\subseteq {\mathcal {E}}_{{\mathcal {N}}}$ if and only if ${\mathcal {M}}\preccurlyeq {\mathcal {N}}$. Thus we see that (M4) means that ${\mathcal {O}}\subsetneq {\mathcal {E}}_{{\mathcal {M}}}$ and (M2) implies that ${\mathcal {E}}_{{\mathcal {M}}}$ is closed under derivation, i.e. if $f\in {\mathcal {E}}_{{\mathcal {M}}}(\Omega )$ then $\partial ^\alpha f\in {\mathcal {E}}_{{\mathcal {M}}}(\Omega )$ for all multi-indices $\alpha \in {\mathbb {N}}_0^n$. Furthermore, we have

### Lemma 2.4

(c.f. Remark 2.5 in [14]) Let the Denjoy–Carleman class ${\mathcal {E}}_{{\mathcal {M}}}$ be closed under derivation closed and suppose that $f\in {\mathcal {E}}_{\mathcal {M}}(\Omega )$ and $f(x_1,\cdots ,x_{j-1},a,x_{j+1},\cdots ,x_n)=0$ for some fixed $a\in {\mathbb {R}}$ and all $x_k$, $k\ne j$, with the property that $(x_1,\cdots ,x_{j-1},a,x_{j+1},\cdots ,x_n)\in \Omega$. Then there exists some $g\in {\mathcal {E}}_{{\mathcal {M}}}(\Omega )$ such that

$$\begin{aligned} f(x)=(x_j -a) g(x). \end{aligned}$$

In fact, if ${\mathcal {M}}$ is a regular weight sequence then the associated Denjoy–Carleman class satisfies a number of further stability properties, see e.g. [4]. In particular, if $f\in {\mathcal {E}}_{{\mathcal {M}}}$ and *G* is a mapping of class $\{{\mathcal {M}}\}$ then $1/f\in {\mathcal {E}}_{{\mathcal {M}}}$ and $f\circ G\in {\mathcal {E}}_{{\mathcal {M}}}$ whenever defined. Furthermore, the inverse function and implicit function theorems hold in the category of regular Denjoy–Carleman classes.

Therefore, we can define manifolds, vector bundles, vector fields, differential forms of class $\{{\mathcal {M}}\}$ as in the smooth (or analytic) category.

### Remark 2.5

Let *E* be an ultradifferentiable vector bundle of class $\{{\mathcal {M}}\}$. Then *E* can also be considered as a smooth vector bundle or as a vector bundle of class ${\mathcal {N}}$ for any weight sequence ${\mathcal {N}}\succcurlyeq {\mathcal {M}}$. We observe in particular that a local basis of ${\mathcal {E}}_{\mathcal {M}}(M,E)$ is also a local basis of ${\mathcal {E}}_{{\mathcal {N}}}(M,E)$ and ${\mathcal {E}}(M,E)$, respectively.

We denote by ${\mathfrak {X}}_{\mathcal {M}}(M)={\mathcal {E}}_{\mathcal {M}}(M, TM)$ the Lie algebra of ultradifferentiable vector fields on *M*. Note that ${\mathcal {E}}_{{\mathcal {M}}}$ is closed under solving ODEs if ${\mathcal {M}}$ is a regular weight sequence, see [22] and [38], and therefore an integral curve of an ultradifferentiable vector field of class $\{{\mathcal {M}}\}$ is an ${\mathcal {E}}_{\mathcal {M}}$-curve.

The next result is the ultradifferentiable version of Sussmann’s Theorem [33].

### Theorem 2.6

Let $p_0\in \Omega$ and a collection ${\mathfrak {D}}$ of ultradifferentiable vector fields of class $\{{\mathcal {M}}\}$. Then there exists an ultradifferentiable submanifold *W* of $\Omega$ through $p_0$ such that all vector fields in ${\mathfrak {D}}$ are tangent to *W* at all points of *W* and such that the following holds:

1. The germ of *W* at $p_0$ is unique, i.e. if $W^\prime$ is an ultradifferentiable submanifold of $\Omega$ containing $p_0$ and to which all vector fields of ${\mathfrak {D}}$ are tangent at every point of $W^\prime$ then there is a neighbourhood $V\subseteq \Omega$ of $p_0$ such that $W\cap V\subseteq W^\prime \cap V$.
2. For every open set $U\subseteq \Omega$ containing $p_0$, there exists $J\in {\mathbb {N}}$ and open neighbourhoods $V_1\subseteq V_2\subseteq U$ of $p_0$ such that every point $p\in W\cap V_1$ can be reached from $p_0$ by a polygonal path of *J* integral curves of vector fields in ${\mathfrak {D}}$ contained in $W\cap V_2$.

The proof of Theorem 2.6 is essentially the same as in the smooth setting, c.f. e.g. [2], due to the fact that the solution of a (Banach space valued) ODE with ultradifferentiable data depends ultradifferentiable on said data, c.f. [38]. The (unique) germ of the manifold *W* will be denoted as the local Sussmann orbit of $p_0$ relative to ${\mathfrak {D}}$. The local Sussmann orbit does not depend on $\Omega$.

One of the main differences between the space of smooth functions and the space of real-analytic functions is that in the smooth case there exist nontrivial test functions $\varphi \in {\mathcal {D}}(\Omega )$, whereas ${\mathcal {D}}\cap {\mathcal {O}}=\{0\}$. Since the existence of nontrivial test functions is equivalent to the existence of nonzero flat functions, it makes sense to give the following definition in the ultradifferentiable setting.

### Definition 2.7

Let $E\subseteq {\mathcal {E}}(\Omega )$ be a subalgebra. We say that *E* is quasianalytic iff for $f\in E$ the fact that $D^\alpha f(p)=0$ for some $p\in \Omega$ and all $\alpha \in {\mathbb {N}}_0^n$ implies that $f\equiv 0$ in the connected component of $\Omega$ that contains *p*.

In the case of Denjoy–Carleman classes, quasianalyticity is characterised by the following theorem.

### Theorem 2.8

(Denjoy [9]–Carleman [6, 7]) The space ${\mathcal {E}}_{\mathcal {M}}(\Omega )$ is quasianalytic if and only if

2.2 $$\begin{aligned} \sum _{k=1}^\infty \frac{m_{k-1}}{km_k}=\infty . \end{aligned}$$

We say that a weight sequence is quasianalytic if it satisfies (2.2) and nonquasianalytic if not.

### Example 2.9

Let $\sigma >0$ be a parameter. We define a family ${\mathcal {N}}^\sigma$ of normal weight sequences by $n_0^\sigma =n_1^\sigma =1$ and

$$\begin{aligned} n_k^\sigma =\bigl (\log (k+e )\bigr )^{\sigma k} \end{aligned}$$

for $k\ge 2$. The weight sequence ${\mathcal {N}}^\sigma$ is quasianalytic if and only if $0<\sigma \le 1$, see [35].

If ${\mathcal {M}}$ is a quasianalytic regular weight sequence then it is possible to show a quasianalytic version of Nagano’s theorem [27], c.f. [14]. As in the case of the ultradifferentiable version of Sussmann’s theorem, the proof is just a straightforward adaptation of the proof of the classical result, see e.g. [2].

### Theorem 2.10

Let *U* be an open neighbourhood of $p_0\in {\mathbb {R}}^n$ and ${\mathcal {M}}$ a quasianalytic regular weight sequence. Furthermore, let ${\mathfrak {g}}$ be a Lie subalgebra of ${\mathfrak {X}}_{\mathcal {M}}(U)$ that is also an ${\mathcal {E}}_{\mathcal {M}}$-module, i.e. if $X\in {\mathfrak {g}}$ and $f\in {\mathcal {E}}_{\mathcal {M}}(U)$ then $fX\in {\mathfrak {g}}$.

Then there exists an ultradifferentiable submanifold *W* of class $\{{\mathcal {M}}\}$ in *U*, such that

2.3 $$\begin{aligned} T_pW={\mathfrak {g}}(p)\qquad \forall p\in W. \end{aligned}$$

Moreover, the germ of *W* at $p_0$ is uniquely defined by this property.

As in the analytic category, c.f. [2], we have the following result.

### Corollary 2.11

Let ${\mathcal {M}}$ be quasianalytic and ${\mathfrak {D}}\subseteq {\mathfrak {X}}_{\mathcal {M}}(\Omega )$ a collection of ultradifferentiable vector fields. If ${\mathfrak {g}}={\mathfrak {g}}_{\mathfrak {D}}$ is the Lie algebra generated by ${\mathfrak {D}}$ and $p_0\in \Omega$ then the local Sussmann orbit of $p_0$, relative to ${\mathfrak {D}}$, coincides with the local Nagano leaf of ${\mathfrak {g}}$.

### Proof

Let $W_N$ be a representative of the local Nagano leaf of ${\mathfrak {g}}$ at $p_0$ and $W_S$ a representative of the local Sussmann orbit of $p_0$, relative to ${\mathfrak {D}}$. By Theorem 2.6 (1) there exists an open neighbourhood *V* of $p_0$ such that $W_S\cap V\subseteq W_N\cap V$. On the other hand ${\mathfrak {g}}(p)=T_pW_N$ for all $p\in W_N$ and ${\mathfrak {g}}(p)\subseteq T_pW_S$ at every $p\in W_S$, hence ${\mathfrak {g}}(p)=T_p W_S$ for $p\in W_S\cap V$. The uniqueness part of Theorem 2.10 gives the equality of the local Nagano leaf and the local Sussmann orbit. $\square$

We want to close this section by showing how the results pertaining the division of smooth functions in [15, section 4] transfer to the category of ultradifferentiable functions of class $\{{\mathcal {M}}\}$. This is possible because these classes are closed under division by a coordinate, i.e. Lemma 2.4 holds.

### Proposition 2.12

Let $U\subseteq {\mathbb {R}}^n$ be a neighbourhood of 0, $\lambda \in {\mathcal {E}}_{\mathcal {M}}(U)$ and suppose that $\lambda$ is of the form $\lambda (x)=x^\alpha {\tilde{\lambda }}(x)$ where $\alpha \in {\mathbb {N}}_0^n$ and ${\tilde{\lambda }}\in {\mathcal {E}}_{\mathcal {M}}(U)$ with ${\tilde{\lambda }}(0)\ne 0$.

If *u* is a locally integrable function near 0 with the property that the product $f:=\lambda \cdot u$ is of class $\{{\mathcal {M}}\}$ near the origin, then *u* is also ultradifferentiable near 0.

### Proof

First, suppose that $\alpha =k e_j$ where $k\in {\mathbb {N}}$ and $e_j$ is the *j*-th unit vector. W.l.o.g. we may assume that $j=n$.

Using Lemma 2.4 we conclude that on some neighbourhood *V* of the origin there is $f_1\in {\mathcal {E}}_{{\mathcal {M}}}(V)$ such that $f(x^\prime ,x_n)=x_nf_1(x^\prime ,x_n)$ on *V*. We want to show that $f_1(x^\prime ,0)=0$ for $(x^\prime ,0)\in V$ if $k>1$: Suppose that there exists some $y\in {\mathbb {R}}^{n-1}$ with $(y,0)\in V$ and $f_1(y,0)\ne 0$. Then there is a neighbourhood *W* of (*y*, 0) such that $f_1(x)\ne 0$ and also ${\tilde{\lambda }}(x)\ne 0$ for $x\in W$. W.l.o.g. the open set *W* is of the form $W=W^\prime \times I\subseteq {\mathbb {R}}^{n-1}\times {\mathbb {R}}$ and we set

$$\begin{aligned} F(x_n):=\int _{W^\prime }\biggl |\frac{f_1}{{\tilde{\lambda }}}(x)\biggr |\,dx \end{aligned}$$

for $x_n\in I$. We conclude that

$$\begin{aligned} \int _W|u(x)|\,dx=\int _I|x_n|^{1-k}F(x_n)\,dx=\infty \end{aligned}$$

and hence *u* cannot be locally integrable near (*y*, 0) which contradicts our assumption. Therefore, we obtain by iteration a function ${\tilde{f}}$ of class $\{{\mathcal {M}}\}$ defined near the origin in ${\mathbb {R}}^n$ such that $f(x^\prime ,x_n)=x_n^k{\tilde{f}}(x^\prime ,x_n)$. Hence $u={\tilde{f}}/{\tilde{\lambda }}$ is also of class $\{{\mathcal {M}}\}$ in a neighbourhood of 0.

In the general case, we argue as follows: Set ${\tilde{f}}=f/{\tilde{\lambda }}$ and

$$\begin{aligned} u_k(x)=\prod _{j=k+1}^n x_j^{\alpha _j} u(x) \end{aligned}$$

for all $k\in \{1,\cdots ,n-1\}$. The function ${\tilde{f}}$ is of class $\{{\mathcal {M}}\}$ whereas the functions $u_k$ are locally integrable near 0. Furthermore, we define $u_{n}=u$ and obtain

$$\begin{aligned} x_1^{\alpha _1}u_1(x)&={\tilde{f}}(x)\\ x_{k+1}^{\alpha _{k+1}}u_{k+1}(x)&=u_{k}(x)\qquad 1\le k\le n-1. \end{aligned}$$

Hence repeated application of the first part finishes the proof. $\square$

## Almost-Analytic Extensions and the Wavefront Set in the Ultradifferentiable Setting

In this section, we recall the almost-analytic extension of ultradifferentiable functions given by Dyn’kin in [10, 11] and the ultradifferentiable wavefront set introduced by Hörmander in [17]. The connection between the both was proven in [14].

We recall (see e.g. [36]) that a smooth function *F* given on an open subset ${\tilde{\Omega }}\subseteq {\mathbb {C}}^n$ is almost analytic iff

$$\begin{aligned} {\bar{\partial }}_jF=\frac{\partial }{\partial {\bar{z}}_j}F=\frac{1}{2}\biggl (\frac{\partial }{\partial x_j}+i\frac{\partial }{\partial y_j}\biggr )F \end{aligned}$$

is flat on ${\tilde{\Omega }}\cap {\mathbb {R}}^n$. The motivation to consider almost-analytic functions in the ultradifferentiable setting is the well-known fact that a function *f* is smooth on $\Omega$ if and only if there is an almost-analytic function *F* on some open set ${\tilde{\Omega }}\subseteq {\mathbb {C}}^n$ with ${\tilde{\Omega }}\cap {\mathbb {R}}^n=\Omega$ such that $F\vert _\Omega =f$. In the ultradifferentiable category, the idea is now that if *f* is ultradifferentiable of class $\{{\mathcal {M}}\}$ then it should be possible to construct an almost-analytic extension *F* of *f* such that the decrease of ${\bar{\partial }}_jF$ can be measured in terms of the weight sequence ${\mathcal {M}}$ (c.f. [12]).

In order to specify this decay, we introduce for a regular weight sequence ${\mathcal {M}}$ its associated weight given by

3.1 $$ \begin{aligned} h_{\mathcal {M}}(t)=\inf _k t^km_k \quad \text {if } t>0\quad { \& }\quad h_{\mathcal {M}}(0)=0. \end{aligned}$$

Conversely, it is possible to extract the weight sequence from its weight, i.e.

$$\begin{aligned} m_k=\sup _t\frac{h_{\mathcal {M}}(t)}{t^k}. \end{aligned}$$

The weight $h_{\mathcal {M}}$ is continuous with values in [0, 1], equals 1 on $[1,\infty )$ and goes more rapidly to 0 than $t^p$ for any $p>0$ for $t\rightarrow 0$, c.f. [14]. Recall also that if $K\subseteq \Omega$ is compact then ${\mathcal {E}}_{{\mathcal {M}}}(K)$ is the space of smooth functions which are defined on some neighbourhood of *K* and on *K* they satisfy (2.1) for some constants $C,h>0$, (c.f. [21]). We are now able to state the main result of [11].

### Theorem 3.1

Let ${\mathcal {M}}$ be a regular weight sequence, $K\subset \subset {\mathbb {R}}^n$ a compact and convex set with $K=\overline{K^\circ }$. Then $f\in {\mathcal {E}}_{\mathcal {M}}(K)$ if and only if there exists a test function $F\in {\mathcal {D}}({\mathbb {C}}^n)$ with $F|_K=f$ and

3.2 $$\begin{aligned} \bigl |{\bar{\partial }}_jF(z,{\bar{z}})\bigr |\le C h_{\mathcal {M}}(Qd_K(z)) \end{aligned}$$

where $1\le j\le n$ and $d_K$ is the distance function with respect to *K* on ${\mathbb {C}}^n\setminus K$.

We call such function *F* an ${\mathcal {M}}$-almost-analytic extension of *f*.

The following theorem is the ${\mathcal {M}}$-almost-analytic version of the almost-holomorphic implicit function theorem proven in [24]. The proof is virtually the same as in the smooth case.

### Theorem 3.2

Let ${\mathcal {M}}$ be a regular weight sequence, $U\subseteq {\mathbb {C}}^N$ a neighbourhood of the origin, $A\in {\mathbb {C}}^p$ and $F:U\times {\mathbb {C}}^p\rightarrow {\mathbb {C}}^N$ of class $\{{\mathcal {M}}\}$ on *U* and polynomial in the last variable with $F(0,A)=0$ and $F_Z(0,A)$ is invertible. Then there exists a neighbourhood $U^\prime \times V^\prime$ of (0, *A*) and a smooth mapping $\phi =(\phi _1,\cdots ,\phi _N): U^\prime \times V^\prime \rightarrow {\mathbb {C}}^N$ with $\phi (0,A)=0$ with the property that if $F(Z,{\bar{Z}},W)=0$ for some $(Z,W)\in U^\prime \times V^\prime$ then $Z=\phi (Z,{\bar{Z}},W)$. Furthermore, there are constants $C,\gamma >0$ such that

3.3 $$\begin{aligned} \biggl |\frac{\partial \phi _j}{\partial Z_k}(Z,{\bar{Z}},W)\biggr |\le Ch_{\mathcal {M}}\bigl (\gamma |\phi (Z,{\bar{Z}},W)- Z|\bigr ) \end{aligned}$$

for all $1\le j,k\le N$ and $\phi$ is holomorphic in *W*.

In the following, we recall the results on the ultradifferentiable wavefront set that we need in this paper. We start with the definition given in [17].

### Definition 3.3

Let ${\mathcal {M}}$ be a regular weight sequence, $u\in {\mathcal {D}}^\prime (\Omega )$ and $(x_0,\xi _0)\in T^*\Omega \setminus \{ 0\}$. We say that *u* is microlocally ultradifferentiable of class $\{{\mathcal {M}}\}$ at $(x_0,\xi _0)$ iff there is a bounded sequence $(u_N)_N\subseteq {\mathcal {E}}^\prime (\Omega )$ such that $u_N\vert _V\equiv u\vert _V$, where *V* is a neighbourhood of $(x_0)$, and a conic neighbourhood $\Gamma$ of $\xi _0$ such that for some constant $Q>0$

3.4 $$\begin{aligned} \sup _{\begin{array}{c} \xi \in \Gamma \\ N\in {\mathbb {N}}_0 \end{array}} \frac{|\xi |^N|{\hat{u}}_N|}{Q^N m_N N!}<\infty . \end{aligned}$$

The ultradifferentiable wavefront set ${{\,\mathrm{WF}\,}}_{\mathcal {M}}u$ is then defined as

$$\begin{aligned} {{\,\mathrm{WF}\,}}_{\mathcal {M}}u:=\bigl \{(x,\xi )\in T^*\Omega \setminus \{ 0\}\mid u\text { is not microlocally ultradiff. of class }\{{\mathcal {M}}\} \text { at }(x,\xi )\bigr \}. \end{aligned}$$

We refer to [19] for the basic properties of ${{\,\mathrm{WF}\,}}_{\mathcal {M}}u$. We shall just mention three facts, that turn out to be important later on. First, if $(p,\xi )\notin {{\,\mathrm{WF}\,}}_{{\mathcal {M}}}u$ for all $\xi \in {\mathbb {R}}^n\setminus \{0 \}$ then *u* is of class $\{{\mathcal {M}}\}$ near *p*. If $P=\sum p_\alpha D^\alpha$ is a linear partial differential operator with ${\mathcal {E}}_{{\mathcal {M}}}$-coefficients, then

3.5 $$\begin{aligned} {{\,\mathrm{WF}\,}}_{{\mathcal {M}}}Pu\subseteq {{\,\mathrm{WF}\,}}_{{\mathcal {M}}}u. \end{aligned}$$

Additionally, we note that ${{\,\mathrm{WF}\,}}_{\mathcal {M}}u$ satisfies the following microlocal reflection property:

3.6 $$\begin{aligned} (x,\xi )\notin {{\,\mathrm{WF}\,}}_{\mathcal {M}}u \Longleftrightarrow (x,-\xi )\notin {{\,\mathrm{WF}\,}}_{\mathcal {M}}{\bar{u}}. \end{aligned}$$

In particular, if *u* is a real-valued distribution, i.e. ${\bar{u}}=u$, then ${{\,\mathrm{WF}\,}}_{\mathcal {M}}u\vert _{x}:=\{\xi \in {\mathbb {R}}^n\mid (x,\xi )\in {{\,\mathrm{WF}\,}}_{\mathcal {M}}u\}$ is symmetric at the origin.

It is a classic fact that the analytic wavefront set can not only be characterised by the Fourier transform but also by holomorphic extensions in certain directions, see [5]. Likewise, the smooth wavefront set can be characterised by almost-analytic extensions, c.f. [26].

In [14], we showed that the ultradifferentiable wavefront set can be characterised by ${\mathcal {M}}$-almost-analytic extensions. This fact in turn allowed us to show that ${{\,\mathrm{WF}\,}}_{{\mathcal {M}}}u$ can be invariantly defined for distributions on manifolds of class $\{{\mathcal {M}}\}$.

In the remainder of this section, we want to recall two theorems from [14], which are crucial for the proof of Theorem 1.1. In order to state the first theorem, we need to recall some notations from [14], which will be also used in the proof of Theorem 1.1.

A subset $\Gamma \subseteq {\mathbb {R}}^d$ is a cone iff for all $\lambda >0$ and $y\in \Gamma$, we have $\lambda y\in \Gamma$. If $r>0$ then

$$\begin{aligned} \Gamma _r :=\bigl \{y\in \Gamma \mid \, |y|<r\bigr \}. \end{aligned}$$

If $\Gamma ^{\prime }\subseteq \Gamma$ is also a cone, we write $\Gamma ^{\prime }\subset \subset \Gamma$ iff $(\Gamma ^{\prime }\cap S^{d-1})\subset \subset (\Gamma \cap S^{d-1})$.

Inspired by [26, section 2.1] (c.f. also [24, section 2]) in the smooth category, we introduce the following notation. If ${\mathcal {M}}$ is a regular weight sequence with associated weight $h_{\mathcal {M}}$, then a function $F\in {\mathcal {E}}(\Omega \times U\times \Gamma _r)$, $U\subseteq {\mathbb {R}}^d$ open is said to be ${\mathcal {M}}$-almost- analytic in the variables $(x,y)\in U\times \Gamma _r$ with parameter $x^\prime \in \Omega$ iff for all $K\subset \subset \Omega$, $L\subset \subset U$ and cones $\Gamma ^{\prime }\subset \subset \Gamma$ there are constants $C,Q>0$ such that for some $r^\prime$ we have

3.7 $$\begin{aligned} \biggl |\frac{\partial F}{\partial {\bar{z}}_j}(x^\prime ,x,y)\biggr |\le Ch_{\mathcal {M}}(Q|y|) \qquad (x^\prime ,x,y)\in K\times L\times \Gamma ^{\prime }_{r^\prime },\;j=1,\cdots ,d.\nonumber \\ \end{aligned}$$

We may also say generally that a function $g\in {\mathcal {C}}(\Omega \times U\times \Gamma _r)$ is of slow growth in $y\in \Gamma _r$ if for all $K\subset \subset \Omega$, $L\subset \subset U$ and $\Gamma ^\prime \subset \subset \Gamma$ there are constants $c,k>0$ such that

3.8 $$\begin{aligned} |g(x^\prime ,x,y)|\le c |y|^{-k} \qquad (x^\prime ,x,y)\in K\times L\times \Gamma ^{\prime }_r. \end{aligned}$$

### Theorem 3.4

Let $F\in {\mathcal {E}}(\Omega \times U\times \Gamma _r)$ be ${\mathcal {M}}$-almost analytic in the variables $(x,y)\in U\times \Gamma _r$ and of slow growth in the variable $y\in \Gamma _r$. Then the distributional limit *u* of the sequence $u_\varepsilon =F(\,.\,,\,.\,,\varepsilon )\in {\mathcal {E}}(\Omega \times U)$ exists. We say that $u=b_\Gamma (F)\in {\mathcal {D}}^\prime (\Omega \times U)$ is the boundary value of *F*. Furthermore, we have

$$\begin{aligned} {{\,\mathrm{WF}\,}}_{\mathcal {M}}u\subseteq \,\bigr (\Omega \times U\bigr )\times \bigl ({\mathbb {R}}^n\times \Gamma ^\circ \bigr ) \end{aligned}$$

where $\Gamma ^\circ =\{\eta \in {\mathbb {R}}^d\mid \langle y,\eta \rangle \ge 0 \;\;\forall y\in \Gamma \}$ is the dual cone of $\Gamma$ in ${\mathbb {R}}^d$.

We close this section by recalling the last fact that we need from [14], the elliptic regularity theorem for partial differential operators with ultradifferentiable coefficients. More precisely we formulate the theorem for differential operators of class $\{{\mathcal {M}}\}$ acting on distributions with values in ultradifferentiable vector bundles. We refer to [8] for more details. We recall here just two details for the convenience of the reader. If *u* is a distribution on an ultradifferentiable manifold *M* of class $\{{\mathcal {M}}\}$ with values in an ${\mathcal {E}}_{{\mathcal {M}}}$-vector bundle over *M*, then we can write locally $u\vert _V=\sum _{j=1}^N u_j\omega ^j$, where the sections $\omega ^1,\cdots ,\omega ^N\in {\mathcal {E}}_{{\mathcal {M}}}(V,E\vert _V)$ constitute a local basis of ${\mathcal {E}}_{\mathcal {M}}(M,E)$. The ultradifferentiable wavefront set of *u* is then defined locally by

$$\begin{aligned} {{\,\mathrm{WF}\,}}_{{\mathcal {M}}}u=\bigcup _{j=1}^N {{\,\mathrm{WF}\,}}_{{\mathcal {M}}}u_j. \end{aligned}$$

On the other hand, if *M* is an manifold of class $\{{\mathcal {M}}\}$, *E* and *F* are two ${\mathcal {E}}_{{\mathcal {M}}}$-vector bundles with the same fibre dimension and $P:{\mathcal {E}}_{{\mathcal {M}}}(M,E)\rightarrow {\mathcal {E}}_{{\mathcal {M}}}(M,F)$ is a partial differential operator of class $\{{\mathcal {M}}\}$ then the principal symbol *p* of *P* is an ultradifferentiable mapping on $T^*M$ with values in the fibre-linear maps from *E* to *F*. The operator *P* is said to be not characteristic (or noncharacteristic) at a point $(x,\xi )\in T^*M\setminus \{0\}$ if $p(x,\xi )$ is an invertible linear mapping. The set of all characteristic points is defined by

$$\begin{aligned} {{\,\mathrm{Char}\,}}P=\{(x,\xi )\in T^{*}M\setminus \{0\}\,:P\text { is characteristic at }(x,\xi )\}. \end{aligned}$$

### Theorem 3.5

Let ${\mathcal {M}}$ be a normal weight sequence, *M* an ultradifferentiable manifold of class $\{{\mathcal {M}}\}$ and *E*, *F* two ultradifferentiable vector bundles on *M* of the same fibre dimension. If *P*(*x*, *D*) is a partial differential operator of class $\{{\mathcal {M}}\}$ between *E* and *F* then

3.9 $$\begin{aligned} {{\,\mathrm{WF}\,}}_{{\mathcal {M}}}u\subseteq {{\,\mathrm{WF}\,}}_{\mathcal {M}}(Pu)\cup \mathrm {Char\,}P\qquad u\in {\mathcal {D}}^{\prime }(M,E). \end{aligned}$$

## CR Manifolds of Denjoy Carleman Type

Recall that an abstract CR manifold $(M,{\mathcal {V}})$ is a smooth manifold *M* with a subbundle ${\mathcal {V}}\subseteq {\mathbb {C}}TM$, called the CR bundle, that is formally integrable, i.e. $[{\mathcal {V}},{\mathcal {V}}]\subseteq {\mathcal {V}}$ and such that ${\mathcal {V}}\cap {\overline{{\mathcal {V}}}}=\{0\}$. The CR dimension of *M* is $\dim _{\mathbb {C}}{\mathcal {V}}$. Important examples of CR manifolds are generic submanifolds of ${\mathbb {C}}^N$. For a generic submanifold $M\subseteq {\mathbb {C}}^N$ the CR bundle ${\mathcal {V}}$ is given by ${\mathcal {V}}= T^{0,1}{\mathbb {C}}^N\cap {\mathbb {C}}TM$, where $T^{0,1}{\mathbb {C}}^N$ is the complex bundle generated by the vector fields $\partial /\partial {\bar{z}}_j$. We refer to [2] for a detailed account of the theory of CR manifolds. Here we just summarise the notations and basic facts that we need in the following.

The sections of ${\mathcal {V}}$ are the CR vector fields of *M*. A CR function (or CR distribution) is a function (or distribution) on *M* that is annihilated by all CR vector fields. We refer to $T^\prime M:={\mathcal {V}}^\perp$ as the holomorphic cotangent bundle and to its sections as holomorphic forms. The real subbundle $T^0M\subseteq T^\prime M$ that consists of the real dual vectors that vanish on ${\mathcal {V}}\oplus {\bar{{\mathcal {V}}}}$ is called the characteristic bundle of *M* and its sections are the characteristic forms on *M*. Note that if *L* is a CR vector field, we have generally that ${{\,\mathrm{Char}\,}}L\subseteq T^0 M$.

A ${\mathcal {C}}^1$-mapping *H* between two CR manifolds $(M,{\mathcal {V}})$ and $(M^\prime ,{\mathcal {V}}^\prime )$ is CR iff for all $p\in M$ we have $H_*({\mathcal {V}}_p)\subseteq {\mathcal {V}}^\prime _{H(p)}$. Here $H_*$ denotes the tangent map of *H*. If $M^\prime \subseteq {\mathbb {C}}^{N^\prime }$ is an embedded CR submanifold and $Z^\prime =(Z^\prime _1,\cdots , Z^\prime _{N^\prime })$ some set of local holomorphic coordinates in ${\mathbb {C}}^{N^\prime }$ then $H_j= Z^{\prime }_j\circ H$, $1\le j\le N^\prime$ is a CR function on the CR manifold *M* for all $1\le j\le N^\prime$.

We note that, as in the smooth case, c.f. [2], we can choose coordinates such that the defining equation of a generic submanifold is of a special form.

### Proposition 4.1

Let $M\subseteq {\mathbb {C}}^N$ be a generic manifold of class $\{{\mathcal {M}}\}$ of codimension *d* and $p_0\in M$. If *n* denotes the CR dimension of *M* then there are holomorphic coordinates $(z,w)\in {\mathbb {C}}^n\times {\mathbb {C}}^d$ defined near $p_0$ that vanish at $p_0$ and a function $\varphi \in {\mathcal {E}}_{\mathcal {M}}(U\times V,{\mathbb {R}}^d)$ defined on a neighbourhood $U\times V$ of the origin in ${\mathbb {R}}^{2n}\times {\mathbb {R}}^d$ with $\varphi (0)=0$ and $\nabla \varphi (0)=0$, such that near $p_0$ the manifold *M* is given by

4.1 $$\begin{aligned} {{\,\mathrm{Im}\,}}w =\varphi (z,{\bar{z}},{{\,\mathrm{Re}\,}}w). \end{aligned}$$

The proof of Proposition 4.1 is analogous to the proof of the statement in the smooth category, [2, Theorem 1.3.6] since the implicit function theorem holds in regular Denjoy–Carleman classes, c.f. [4].

Next we give a first result on the structure of ultradifferentiable CR manifolds. For that we have to recall some further definitions from [2]. Let $M\subseteq {\mathbb {C}}^N$ be a CR submanifold of class $\{{\mathcal {M}}\}$ and $p\in M$. The CR orbit ${\mathrm {Orb}}_p$ of *p* is the local Sussmann orbit of *p* in *M* relative to the set of ultradifferentiable sections of $T^cM={{\,\mathrm{Re}\,}}{\mathcal {V}}$. Note that if $p\in M$ then by construction $T^c_q{\mathrm {Orb}}_{q}=T^c_pM$ for all $Q\in {\mathrm {Orb}}_{p_0}$ thence ${\mathrm {Orb}}_{p}$ is the germ of a CR submanifold of ${\mathbb {C}}^N$ with CR dimension *n*.

We say that *M* is minimal at $p_0$ iff there is no submanifold $S\subseteq M$ through $p_0$ such that $T^c_p M\subseteq T_p S$ for all $p\in S$ and $\dim _{\mathbb {R}}S <\dim _{\mathbb {R}}M$. The manifold *M* is said to be of finite type at $p_0$ iff there are vector fields $X_1,\cdots ,X_k\in {\mathcal {E}}_{\mathcal {M}}(M, T^cM)$ such that the Lie algebra generated by the $X_1,\cdots ,X_k$ evaluated at $p_0$ is isomorphic to $T_{p_0}M$. It is well known that finite type implies minimality and that the two notions coincide for real-analytic CR manifolds, c.f. [2]. We are going to show that this fact holds also for quasianalytic CR submanifolds.

### Theorem 4.2

Let ${\mathcal {M}}$ be a quasianalytic regular weight sequence and $M\subseteq {\mathbb {C}}^N$ an ultradifferentiable CR manifold of class $\{{\mathcal {M}}\}$. The following statements are equivalent:

1. *M* is minimal at $p_0$.
2. $$\dim _{\mathbb {R}}{\mathrm {Orb}}_{p_0}=\dim _{\mathbb {R}}M$$.
3. *M* is of finite type at $p_0$.

### Proof

The equivalence of (1) and (2) holds even if ${\mathcal {M}}$ is nonquasianalytic. Following the arguments in [2, § 4.1.] we see that, if we assume that *M* is nonminimal then $\dim _{\mathbb {R}}{\mathrm {Orb}}_{p_0}<\dim _{\mathbb {R}}M$. On the other hand, if $\dim _{\mathbb {R}}{\mathrm {Orb}}_{p_0}<\dim _{\mathbb {R}}M$ then any representative *W* of ${\mathrm {Orb}}_{p_0}$ is by the remark above a proper submanifold of *M* and $T^c_p W=T^c_pM$ for all $p\in W$. It remains to prove that (2) implies (3).

By Corollary 2.11, we have that ${\mathrm {Orb}}_{p_0}=\gamma _{p_0}({\mathfrak {g}})$, where ${\mathfrak {g}}$ is the Lie algebra generated by the ultradifferentiable sections of $T^cU$ with *U* being a sufficiently small neighbourhood of $p_0$ and $\gamma _{p_0}({\mathfrak {g}})$ the local Nagano leaf of ${\mathfrak {g}}$ at $p_0$. Hence $\dim _{\mathbb {R}}{\mathrm {Orb}}_{p_0}=\dim _{\mathbb {R}}\gamma _{p_0}({\mathfrak {g}})=\dim _{\mathbb {R}}{\mathfrak {g}}(p_0)$.

On the other hand, *M* is of finite type at $p_0$ if and only if $\dim _{\mathbb {R}}{\mathfrak {g}}(p_0) =\dim _{\mathbb {R}}M$.    $\square$

The next example is a straightforward generalisation of [2, Example 1.5.16].

### Example 4.3

Let ${\mathcal {M}}$ be a nonquasianalytic regular weight sequence and $\psi \in {\mathcal {E}}_{\mathcal {M}}({\mathbb {R}})$ a real-valued function such that $\psi (y)=0$ for $y\le 0$ and $\psi (y)>0$ for $y>0$. We define a real hypersurface in ${\mathbb {C}}^2$ by

$$\begin{aligned} M=\bigl \{(z,w)\in {\mathbb {C}}^2\mid {{\,\mathrm{Im}\,}}w=\varphi ({{\,\mathrm{Im}\,}}z)\bigr \}. \end{aligned}$$

Then *M* is minimal at the origin but not of finite type at 0. Indeed, if *M* is nonminimal at 0 then according to [2, Theorem 1.5.15] there is a holomorphic hypersurface $S\subseteq M$ through the origin. Since $\partial /\partial z$ is tangent to *S* at 0 it follows that *S* is given near the origin by the defining equation $w=h(z)$ where *h* is a holomorphic function defined in some neighbourhood of $0\in {\mathbb {C}}$ with $h(0)=0$. We conclude that due to $S\subseteq M$ we necessarily have that

$$\begin{aligned} h(z)-\overline{h(z)}=2i\psi ({{\,\mathrm{Re}\,}}z) \end{aligned}$$

in some neighbourhood of 0. It follows that $\psi$ has to be real analytic near 0 which contradicts the definition of $\psi$. Since $\psi$ is flat at the origin, it follows that *M* cannot be of finite type at 0.

We close this section by recalling the space of multipliers for an ultradifferentiable abstract CR manifold $(M,{\mathcal {V}})$, which was introduced in [15] for the smooth setting. To begin with, consider the following sequence of spaces of sections

$$\begin{aligned} E_k=\bigl \langle {\mathcal {L}}_{K_1}\dots {\mathcal {L}}_{K_j}\theta :j\le k,\, \, K_q\in {\mathcal {E}}_{\mathcal {M}}(M, {\mathcal {V}}), \, \theta \in {\mathcal {E}}_{\mathcal {M}}(M,T^0 M) \bigr \rangle . \end{aligned}$$

We note that $E_0 = {\mathcal {E}}_{\mathcal {M}}(M, T^0 M)$, and $E_j \subseteq {\mathcal {E}}_{\mathcal {M}}(M, T' M)$ for all $j\in {\mathbb {N}}_0$, and set $E = \bigcup _{j\in {\mathbb {N}}_0} E_j$.

We associate to the increasing chain $E_k$ the increasing sequence of ideals ${\mathcal {S}}^k \subset {\mathcal {E}}_{\mathcal {M}}(M,{\mathbb {C}})$, where

$$\begin{aligned} {\mathcal {S}}^k = \bigwedge \nolimits ^N E_k = \left\{ \det \begin{pmatrix} V^1 ( {\mathfrak {Y}}_1 ) &{} \dots &{} V^1 ( {\mathfrak {Y}}_N ) \\ \vdots &{} &{} \vdots \\ V^N ( {\mathfrak {Y}}_1 ) &{} \dots &{} V^N ( {\mathfrak {Y}}_N ) \\ \end{pmatrix} :V^j \in E_k,\, {\mathfrak {Y}}_j \in {\mathcal {E}}_{\mathcal {M}}(M,(T'M)^*) \right\} . \end{aligned}$$

We set ${\mathcal {S}}={\mathcal {S}}(M)=\bigcup _{k\in {\mathbb {N}}_0} {\mathcal {S}}^k$ and call it the space of multipliers of *M*. In fact each ${\mathcal {S}}^k$ and thus also ${\mathcal {S}}$ can be considered actually as ideal sheaves, if we define $E^k(U)$ and ${\mathcal {S}}^k(U)$ accordingly.

Note that locally one can find smaller sets of generators: Let $U\subseteq M$ be open, and assume that $L_1,\dots ,L_n$ is a local basis for $\Gamma (U,{\mathcal {V}})$, that $\theta ^1, \dots ,\theta ^d$ is a local basis for $\Gamma (U,T^0M)$, and that $\omega ^1, \dots , \omega ^N$ is a local basis of $T^\prime M$. We write ${\mathcal {L}}_j = {\mathcal {L}}_{L_j}$ for $j = 1, \dots , n$ and ${\mathcal {L}}^\alpha = {\mathcal {L}}_1^{\alpha _1} \dots {\mathcal {L}}_n^{\alpha _n}$ for any multi-index $\alpha = (\alpha _1, \dots , \alpha _n)\in {\mathbb {N}}^n$. We note that, since ${\mathcal {V}}$ is formally integrable, the ${\mathcal {L}}^\alpha$, where $|\alpha | = k$, generate all *k*-th-order homogeneous differential operators in the ${\mathcal {L}}_j$, and we thus have

$$\begin{aligned} E_k\big |_U =\bigl \langle {\mathcal {L}}^\alpha \theta ^\mu \,:\;\; 1 \le \mu \le d,\, \, |\alpha | \le k \bigr \rangle . \end{aligned}$$

We can expand

4.2 $$\begin{aligned} {\mathcal {L}}^\alpha \theta ^\mu = \sum _{\ell =1}^{N} A^{\alpha ,\mu }_\ell \omega ^\ell \end{aligned}$$

and for any choice ${{\underline{\alpha }}} = (\alpha ^1, \dots , \alpha ^N )$ of multi-indices $\alpha ^1, \dots , \alpha ^N \in {\mathbb {N}}_0^n$ and $r = (r_1,\dots , r_N) \in \{1,\dots ,d\}^N$ we define the functions

4.3 $$\begin{aligned} D({{\underline{\alpha }}} ,r) = \det \begin{pmatrix} A^{\alpha ^1,r_1}_1 &{} \dots &{} A^{\alpha ^1,r_1}_N \\ \vdots &{} &{} \vdots \\ A^{\alpha ^N,r_N}_1 &{} \dots &{} A^{\alpha ^N,r_N}_N \\ \end{pmatrix} . \end{aligned}$$

With this notation, we have

$$\begin{aligned} {\mathcal {S}}^k \big |_U = \left\langle D({{\underline{\alpha }}} ,r):|\alpha ^j|\le k \right\rangle ; \end{aligned}$$

we shall denote the stalk of ${\mathcal {S}}^k$ at *p* by ${\mathcal {S}}^k_{p}$.

The space of multipliers of a CR manifold *M* clearly encodes the nondegeneracy properties of *M*. We close this section by taking a closer look at the connection of ${\mathcal {S}}$ with finite nondegeneracy. We recall from [2] the definition of finite nondegeneracy for abstract CR manifolds.

### Definition 4.4

Let *M* be an abstract CR manifold and

4.4 $$\begin{aligned} E_k(p)=\bigl \langle {\mathcal {L}}_{K_1}\dots {\mathcal {L}}_{K_j}\theta (p):j\le k,\, \, K_q\in {\mathcal {E}}(M, {\mathcal {V}}), \, \theta \in {\mathcal {E}}(M,T^0 M) \bigr \rangle . \end{aligned}$$

for $p\in M$ and $k\in {\mathbb {N}}$. Then *M* is $k_0$-nondegenerate at $p_0\in M$ iff $E_{k_0-1}\subsetneq E_{k_0}=T^\prime _{p_0}M$. We say that *M* is finite nondegenerate iff *M* is finite nondegenerate at every point.

### Remark 4.5

This definition is in fact local, since by [2, Proposition 11.1.10], if $L_1,\cdots , L_n$ is a local basis of CR vector fields and $\theta ^1,\cdots \theta ^d$ is a local basis of characteristic forms near $p_0$ then *M* is $k_0$-nondegenerate if and only if

$$\begin{aligned} T^\prime _{p_0}M={{\,\mathrm{span}\,}}_{\mathbb {C}}\bigl \{{\mathcal {L}}^\alpha \theta ^{\mu }(p_0):|\alpha |\le k_0,\;\mu \in \{1,\cdots ,d\}\bigr \}. \end{aligned}$$

Hence we may replace *M* with any open neighbourhood $U\subseteq M$ of $p_0$ in (4.4). Thus we observe that a CR submanifold *M* is $k_0$-nondegenerate at $p_0\in M$ if and only if ${\mathcal {S}}^{k_0}_{p_0}=({\mathcal {E}}_{\mathcal {M}})_{p_0}$.

More precisely, let $U\subseteq M$ be an open subset and $q\in U$. Then *M* is $k_0$-nondegenerate at *q* if and only if there is a multiplier $f\in {\mathcal {S}}^{k_0}(U)$ that does not vanish at *q*, i.e. $f(q)\ne 0$. Indeed, if $f(q)\ne 0$ then obviously $E_{k_0}(q)=T^\prime _q M$. On the other hand, if $g(q)=0$ for all multipliers $g\in {\mathcal {S}}^{k_0}(U)$ then necessarily $E_{k_0}(q)\ne T^\prime _{q}M$.

## Ultradifferentiable Regularity of CR Mappings

The main goal of this section is to present the proof of Theorem 1.1. Furthermore, we show also ultradifferentiable versions of further regularity results of [3] and [24]. However, first we need to recall the definition of finite nondegeneracy of a CR mapping.

### Definition 5.1

Let *M* be an abstract CR manifold and $M^\prime \subseteq {\mathbb {C}}^{N^\prime }$ a generic submanifold. Furthermore let $\rho ^\prime =(\rho ^\prime _1,\cdots ,\rho ^\prime _{d^\prime })$ be a defining function of $M^\prime$ near a point $q_0\in M^\prime$, $L_1,\cdots , L_n$ a local basis of CR vector fields on *M* near $p_0\in M$ and $H: M\rightarrow M^\prime$ a ${\mathcal {C}}^m$-CR mapping with $H(p_0)=q_0$.

For $0\le k\le m$ define an increasing sequence of subspaces $E_k(p_0)\subseteq {\mathbb {C}}^{N^\prime }$ by

$$\begin{aligned} E_k(p_0):={{\,\mathrm{span}\,}}_{\mathbb {C}}\biggl \{L^{\alpha }\frac{\partial \rho ^\prime }{\partial Z^\prime } \bigl (H(Z),\overline{H(Z)}\bigr )\vert _{Z=p_0}\;:\; 0\le |\alpha |\le k,\,1\le l\le d^\prime \biggr \}. \end{aligned}$$

We say that *H* is $k_0$-nondegenerate at $p_0$ ($0\le k_0\le m$) iff $E_{k_0-1}(p_0)\subsetneq E_{k_0}(p_0)={\mathbb {C}}^{N^\prime }$.

### Remark 5.2

Comparing Definition 5.1 with Definition 4.4 we observe that a CR submanifold $M\in {\mathbb {C}}^N$ is $k_0$-nondegenerate if and only if ${{\,\mathrm{id}\,}}:\, M\rightarrow M$ is $k_0$-nondegenerate. We note also the fact that any CR diffeomorphism between two $k_0$-nondegenerate CR submanifolds is $k_0$-nondegenerate.

Finally, we need to recall that if $\rho$ is a local defining function of *M*, $\Gamma \subseteq {\mathbb {R}}^d$ an open convex cone, $p_0\in M$ and $U\subseteq {\mathbb {C}}^N$ an open neighbourhood of $p_0$, then a wedge ${\mathcal {W}}$ with edge *M* centred at $p_0$ is an open subset of the form ${\mathcal {W}}:=\{Z\in U\mid \rho (Z,{\bar{Z}})\in \Gamma \}$.

### Proof of Theorem 1.1

Since the assertion of the theorem is local, we are going to work on a neighbourhood $\Omega \subseteq {\mathbb {C}}^N$ of $p_0$. If $\Omega$ is small enough then by Proposition 4.1 there are open neighbourhoods $U\subseteq {\mathbb {C}}^n$ and $V\subseteq {\mathbb {R}}^d$ of the origin and a function $\varphi \in {\mathcal {E}}_{\mathcal {M}}(U\times V,{\mathbb {R}}^d)$ with $\varphi (0,0)=0$ and $\nabla \varphi (0,0)=0$ such that

$$\begin{aligned} M\cap \Omega =\bigl \{(z,w)\in \Omega \mid {{\,\mathrm{Im}\,}}w=\varphi (z,{\bar{z}},{{\,\mathrm{Re}\,}}w)\bigr \}. \end{aligned}$$

From now on we denote $M\cap \Omega$ by *M*. If we choose *U* and *V* to be small enough, we can consider the diffeomorphism

$$\begin{aligned} \Psi :\; U\times V&\longrightarrow \qquad M\\ (z,s)\;&\longmapsto (z,s+i\varphi (z,{\bar{z}},s)). \end{aligned}$$

If we shrink the neighbourhoods *U*, *V* a little bit (such that $$\varphi \in {\mathcal {E}}_{\mathcal {M}}(\overline{U\times V},{\mathbb {R}}^d)$$) and assume that w.l.o.g. both sets are convex then by Theorem 3.1 we can extend the mapping $$\Psi $$$${\mathcal {M}}$$-almost analytically in the *s*-variables , i.e. there exists a smooth function $${\tilde{\Psi }}:\, U\times V\times {\mathbb {R}}^d\rightarrow {\mathbb {C}}^N$$ such that $${\tilde{\Psi }}\vert _{U\times V\times \{0\}}=\Psi $$ and for each component $${\tilde{\Psi }}_k$$, $$k=1,\cdots ,N$$, of $${\tilde{\Psi }}$$ we have

5.1 $$\begin{aligned} \biggl |\frac{\partial {\tilde{\Psi }}_k}{\partial {\bar{w}}^\prime _j}(z,{\bar{z}},s,t)\biggr |\le Ch_{\mathcal {M}}(\gamma |t|)\qquad j=1,\cdots , d, \end{aligned}$$

for some constants $C,\gamma >0$. Here $w^\prime =s+it\in V+i{\mathbb {R}}^d$. We see that there is some $r>0$ such that ${\tilde{\Psi }}\vert _{U\times V\times B_r(0)}$ is a diffeomorphism.

By assumption $H=(H_1,\cdots ,H_{N^{\prime }})$ extends continuously to a holomorphic mapping on a wedge ${\mathcal {W}}$ near 0. If we shrink ${\mathcal {W}}$ we may assume that $\partial H_j$, $j=1,\cdots ,N^\prime$, is bounded on ${\mathcal {W}}$. By definition

$$\begin{aligned} {\mathcal {W}}=\bigl \{Z\in \Omega _0\mid \rho (Z,{\bar{Z}})\in {\tilde{\Gamma }}\bigr \} \end{aligned}$$

for a neighbourhood $\Omega _0$ of the origin in ${\mathbb {C}}^N$ and an open acute cone ${\tilde{\Gamma }}\subseteq {\mathbb {R}}^d$. If we shrink *U*, *V*, when necessary, and choose a suitable open and acute cone $\Gamma$, we achieve that

$$\begin{aligned} {\tilde{\Psi }}\bigl (U\times V\times \Gamma _\delta \bigr )\subseteq {\mathcal {W}}\end{aligned}$$

for some $r\ge \delta >0$. Note that ${\tilde{\Psi }}(U\times V\times \Gamma _\delta )$ is open in ${\mathbb {C}}^N$. For each $j=1,\cdots ,N^\prime$ set $h_j=H_j\circ {\tilde{\Psi }}$ and $u_j=H_j\circ \Psi$. Since

$$\begin{aligned} \frac{\partial h_j}{\partial {\bar{w}}^\prime _k}=\sum _{\ell =1}^N\frac{\partial H_j}{\partial Z_\ell } \frac{\partial {\tilde{\Psi }}_\ell }{\partial {\bar{w}}^\prime _k} \qquad j=1,\cdots , N^\prime ,\; k=1,\cdots , d, \end{aligned}$$

and $\partial H_j$ is bounded, each function $h_j$ is ${\mathcal {M}}$-almost analytic on $U\times V\times \Gamma _\delta$ due to (5.1) and extends $u_j\in {\mathcal {C}}^{k_0}(U\times V)$. Hence Theorem 3.4 implies that

5.2 $$\begin{aligned} {{\,\mathrm{WF}\,}}_{\mathcal {M}}u_j\subseteq \bigl (U\times V\bigr )\times \bigl ({\mathbb {R}}^{2n}\times \Gamma ^\circ \bigr )\setminus \{0\}. \end{aligned}$$

If $L_j\in {\mathcal {E}}_{{\mathcal {M}}}(M,{\mathcal {V}})$, $j=1,\cdots , n$, form a basis of the CR vector fields on $M=M\cap \Omega$, then the vector fields $\Lambda _j=\Psi ^*L_j$ define a CR structure on $U\times V$ and $\Lambda _j u_k=0$ for $j=1,\cdots , n$ and $k=1,\cdots , N^\prime$.

Let $\rho ^\prime$ be a defining function of $M^\prime$ near $p^\prime _0=0\in {\mathbb {C}}^{N^\prime }$. Then there are ultradifferentiable functions $\Phi _{\ell ,\alpha }(Z^\prime ,{\bar{Z}}^\prime ,W)$ for $|\alpha |\le k_0$, $\ell =1,\cdots ,d^\prime$, defined in a neighbourhood of $\{0\}\times {\mathbb {C}}^{K_0}\subseteq {\mathbb {C}}^{N^\prime }\times {\mathbb {C}}^{K_0}$ and polynomial in the last $K_0=N^\prime \cdot |\{\alpha \in {\mathbb {N}}^n_0\mid |\alpha |\le k_0\}|$ variables such that

5.3 $$\begin{aligned} \Lambda ^\alpha \bigl (\rho _{\ell }^\prime \circ u\bigr )(z,{\bar{z}},s)=\Phi _{\ell ,\alpha } \Bigl (u(z,{\bar{z}},s),{\bar{u}}(z,{\bar{z}},s),\bigl (\Lambda ^\beta {\bar{u}}(z,{\bar{z}},s) \bigr )_{|\beta |\le k_0}\Bigr )=0\nonumber \\ \end{aligned}$$

and

$$\begin{aligned} \Lambda ^\alpha \rho ^\prime _{\ell ,Z^\prime }\bigl (u,{\bar{u}}\bigr )(0,0,0) =\Phi _{\ell ,\alpha ,Z^\prime }\bigl (0,0,(\Lambda ^\beta {\bar{u}}(0,0,0))_{|\beta |\le k_0}\bigr ). \end{aligned}$$

Since *H* is $k_0$-nondegenerate there are multi-indices $\alpha ^1,\cdots ,\alpha ^{N^\prime }$ and $\ell ^1,\cdots ,\ell ^{N^\prime }\in \{1,\dots ,d^\prime \}$ such that if we set

$$\begin{aligned} \Phi =\bigl (\Phi _{\ell ^1,\alpha ^1},\cdots ,\Phi _{\ell ^{N^\prime },\alpha ^{N^\prime }}\bigr ) \end{aligned}$$

then the matrix $\Phi _{Z^\prime }$ is invertible. Hence by Theorem 3.2 there is a smooth function $\phi =(\phi _1,\cdots ,\phi _{N^\prime })$ defined in a neighbourhood of $(0,(\Lambda ^\beta {\bar{u}}(0,0,0))_{|\beta |})$ in ${\mathbb {C}}^{N^\prime }\times {\mathbb {C}}^{K_0}$ such that, if we shrink $U\times V$ accordingly,

$$\begin{aligned} u_j(z,{\bar{z}},s)=\phi _j\bigl (u(z,{\bar{z}},s),{\bar{u}}(z,{\bar{z}},s),(\Lambda ^\beta {\bar{u}}(z,{\bar{z}},s))_{|\beta |\le k_0}\bigr )\qquad (z,s)\in U\times V,\;\;j=1,\cdots ,N^\prime \end{aligned}$$

and (3.3) holds. If we further shrink $U\times V$ and $\delta$ and choose $\Gamma ^\prime \subset \subset \Gamma$ appropriately we see that

5.4 $$\begin{aligned} g_j (z,{\bar{z}},s,t)=\phi _j\bigl (h(z,{\bar{z}},s,-t),{\bar{h}}(z,{\bar{z}},s,-t), ({\tilde{h}}_{\ell ,\beta }(z,{\bar{z}},s,t)_{\ell \in \{1,\cdots ,N^\prime \};|\beta |\le k_0}\bigr )\nonumber \\ \end{aligned}$$

is well defined for $t\in -\Gamma ^\prime _\delta$. Here ${\tilde{h}}_{j,\beta }$ is the ${\mathcal {M}}$-almost-analytic extension of $\Lambda ^\beta {\bar{u}}_j$ on $U\times V\times (-\Gamma ^\prime _\delta )$, which exists due to (5.2), (3.6), (3.5) and [14, Theorem 4.4]. It is also easy to see that ${\bar{h}}(z,{\bar{z}},s,-t)$ is ${\mathcal {M}}$-almost analytic on $U\times V\times (-\Gamma ^\prime _\delta )$. We have that

5.5 $$\begin{aligned} \frac{\partial g_j}{\partial {\bar{w}}^\prime _\ell }= \sum _{k=1}^{N^\prime }\frac{\partial \phi _j}{\partial Z^\prime _k} \frac{\partial h_k}{\partial w^\prime _\ell } +\sum _{k=1}^{N^{\prime }} \frac{\partial \phi _j}{\partial {\bar{Z}}^{\prime }}\frac{\partial {\bar{h}}}{\partial w^\prime _\ell } +\sum _{k=1}^{N^\prime }\sum _{|\beta |\le k_0}\frac{\partial \phi _j}{\partial W_{k,\beta }}\frac{\partial {\tilde{h}}_{k,\beta }}{\partial w^\prime _\ell } \end{aligned}$$

for $j=1,\cdots ,N^\prime$ and $\ell =1,\cdots ,d$. Note that we can choose $U\times V$ and $\Gamma ^\prime _\delta$ so small that all functions appearing on the right-hand side are uniformly bounded. Hence, since $\partial _{w_\ell ^\prime }{\bar{h}}=\overline{\partial _{{\bar{w}}^\prime _\ell } h}$, the last two terms on the right-hand side of (5.5) are ${\mathcal {M}}$-almost analytic. The estimate (3.3) and the arguments in [25, Section 3.3] give that the first sum on the right-hand side of (5.5) is also ${\mathcal {M}}$-almost analytic. We conclude that $g_j$ is an ${\mathcal {M}}$-almost analytic extension on $U\times V\times (-\Gamma ^\prime _\delta )$ of $u_j$ and thus

$$\begin{aligned} {{\,\mathrm{WF}\,}}_{\mathcal {M}}u_j\subseteq \bigl (U\times V\bigr )\times \bigl ({\mathbb {R}}^n\times (\Gamma ^\prime \cup -\Gamma ^\prime )^\circ \bigr )\setminus \{0\} =\bigl (U\times V\bigr )\times \bigl ({\mathbb {R}}^n\setminus \{0\}\times \{0\}\bigr ). \end{aligned}$$

On the other hand, since each $u_j$ is CR we have that ${{\,\mathrm{WF}\,}}_{\mathcal {M}}u_j\vert _0\subseteq \{0\}\times {\mathbb {R}}^d\setminus \{0\}$ by (3.9) and we deduce that in fact ${{\,\mathrm{WF}\,}}_{\mathcal {M}}u_j\vert _0=\emptyset$ for all $j=1,\cdots ,N^\prime$. Hence the mapping *H* is ultradifferentiable of class $\{{\mathcal {M}}\}$ near $p_0$. $\square$

In the rest of this section, it is always assumed that the weight sequence ${\mathcal {M}}$ is normal.

If we recall the well-known theorem of Tumanov [37] which states that any CR function on a minimal CR submanifold *M* extends to a holomorphic function on a wedge with edge *M*, then we obtain the following corollary.

### Corollary 5.3

Let $M\subseteq {\mathbb {C}}^N$ and $M^\prime \subseteq {\mathbb {C}}^{N^\prime }$ be generic submanifolds of class $\{{\mathcal {M}}\}$, $p_0\in M$, $p_0^\prime \in M^\prime$, *M* minimal at $p_0$ and $H:\,(M,p_0)\rightarrow (M^\prime ,p_0^\prime )$ a ${\mathcal {C}}^{k_0}$-CR mapping that is $k_0$-nondegenerate at $p_0$. Then *H* is ultradifferentiable of class $\{{\mathcal {M}}\}$ in some neighbourhood of $p_0$.

This leads to the following result.

### Corollary 5.4

Let $M\subseteq {\mathbb {C}}^N$ and $M^\prime \subseteq {\mathbb {C}}^{N^\prime }$ be generic submanifolds of class $\{{\mathcal {M}}\}$ that are both $k_0$-nondegenerate at $p_0\in M$ and $p_0^\prime \in M^\prime$, respectively. Furthermore assume that *M* is minimal at $p_0$ and let $H: M\rightarrow M^\prime$ a CR diffeomorphism that is ${\mathcal {C}}^{k_0}$ near $p_0$ and satisfies $H(p_0)=p_0^\prime$. Then *H* has to be ultradifferentiable of class $\{{\mathcal {M}}\}$ near $p_0$.

Recently, Berhanu–Xiao [3] showed that it is possible to slightly weaken the prerequisites of the smooth regularity result of Lamel. In particular, the source manifold *M* can be chosen to be an abstract CR manifold. Using the methods developed previously we can also generalise this result to the ultradifferentiable category.

### Theorem 5.5

Let $(M,{\mathcal {V}})$ be an abstract CR manifold and $M^\prime \subseteq {\mathbb {C}}^{N^\prime }$ be a generic submanifold, both of class $\{{\mathcal {M}}\}$. Furthermore let $p_0\in M$, $H:\,M\rightarrow M^\prime$ a ${\mathcal {C}}^{k_0}$-CR mapping that is $k_0$-nondegenerate at $p_0$ and there is a closed acute cone $\Gamma \subseteq {\mathbb {R}}^d$ such that ${{\,\mathrm{WF}\,}}_{\mathcal {M}}H\vert _{p_0}\subseteq \{0\}\times \Gamma$. Then *H* is ultradifferentiable of class $\{{\mathcal {M}}\}$ near $p_0$.

### Proof

Since the assertion is local we will work on a small chart neighbourhood $\Omega =U\times V\times W\subseteq {\mathbb {R}}^n\times {\mathbb {R}}^n\times {\mathbb {R}}^d$ of *M* of $p_0=0$. Here *n* denotes the CR dimension of *M*, whereas *d* is the CR codimension of *M*. We use coordinates (*x*, *y*, *s*) on $\Omega$ and write $z=x+iy$. In these coordinates a local basis of the CR vector fields of *M* is given by

$$\begin{aligned} L_j=\frac{\partial }{\partial {\bar{z}}_j}+\sum _{k=1}^n a_{jk}\frac{\partial }{\partial z_k} +\sum _{\alpha =1}^db_{j\alpha }\frac{\partial }{\partial s_\alpha }\qquad j=1,\cdots ,n. \end{aligned}$$

From the assumptions we conclude that if $\Omega$ is small enough that there is an open convex cone $\Gamma _1\subseteq {\mathbb {R}}^{N}\setminus \{0\}$ such that

5.6 $$\begin{aligned} {{\,\mathrm{WF}\,}}_{\mathcal {M}}H=\bigcup _{j=1}^{N^\prime }{{\,\mathrm{WF}\,}}_{\mathcal {M}}H_j\subseteq \Omega \times \Gamma _1^\circ \end{aligned}$$

due to the closedness of ${{\,\mathrm{WF}\,}}_{\mathcal {M}}H$ in $T^{*}M\setminus \{0\}$. If we further shrink $\Omega$ (resp. *U*, *V* and *W*) and choose an open convex cone $\Gamma _2\subseteq {\mathbb {R}}^{N}\setminus \{0\}$ such that ${\overline{\Gamma }}_2\subseteq \Gamma _1\cup \{0\}$ we have by [14, Theorem 4.4] that there is an ${\mathcal {M}}$-almost extension ${\tilde{F}}$ with slow growth of *H* onto $\Omega \times \Gamma _2$. If we now choose an open convex cone $\Gamma _3\subseteq {\mathbb {R}}^d\setminus \{0\}$ with $\{0\}\times \Gamma _3\subseteq \Gamma _2$, we infer that

$$\begin{aligned} F:={\tilde{F}}\vert _{\Omega \times (\{0\}\times \Gamma _3)} \end{aligned}$$

is an ${\mathcal {M}}$-almost-analytic function on $U\times V\times W\times \Gamma _3$ with values in ${\mathbb {C}}^{N^\prime }$ and

$$\begin{aligned} \lim _{\Gamma _3\ni t\rightarrow 0} F(\,.\,,\,.\,,\,.\,,t)=H \end{aligned}$$

in the sense of distributions.

Let $\rho ^\prime =(\rho _1^\prime ,\cdots ,\rho _{N^\prime }^\prime )$ be an ultradifferentiable defining function of $M^\prime$ near $p^\prime _0=H(p_0)$. As before in the proof of Theorem 1.1 we conclude that there are ultradifferentiable functions $\Phi _{\ell ,\alpha }(Z^\prime ,{\bar{Z}}^\prime , W)$ for $|\alpha |\le k_0$, $\ell =1,\cdots ,d^\prime$, defined in a neighbourhood of $\{0\}\times {\mathbb {C}}^{K_0}\subset {\mathbb {C}}^{N^\prime }\times {\mathbb {C}}^{K_0}$ and polynomial in the last $K_0=N^\prime |\{\alpha \in {\mathbb {N}}^{n^\prime }_0\mid |\alpha |\le k_0\}|$ variables. From now on we can follow the proof of Theorem 1.1 verbatim. $\square$

## Ultradifferentiable Regularity of Infinitesimal CR Automorphisms

In this section, we show how the results in [15] concerning the smoothness of infinitesimal CR automorphisms transfer to the ultradifferentiable setting. Since our presentation here differs in some details from that given in [15], we first recall the framework we are going to work in. In this section, $(M,{\mathcal {V}})$ is always an ultradifferentiable abstract CR manifold of class $\{{\mathcal {M}}\}$ with ${\mathcal {M}}$ being a normal weight sequence.

### Definition 6.1

Let $U\subseteq M$ an open subset and $X: U\rightarrow TM$ a vector field of class ${\mathcal {C}}^1$. We say that *X* is an infinitesimal CR automorphism iff its flow $H^\tau$, defined for small $\tau$, has the property, that there is $\varepsilon >0$ such that $H^\tau$ is a CR mapping provided that $|\tau |\le \varepsilon$.

We need for the proofs of the regularity results a more suitable characterisation of infinitesimal CR automorphisms. We call a section ${\mathfrak {Y}}\in \Gamma (M,(T^\prime M)^*)$ a holomorphic vector field on *M*.

Apparently every vector field $X\in \Gamma (M,TM)$ gives rise to a holomorphic vector field by first extending *X* to ${\mathbb {C}}TM$ and then restricting the extension to $T^*M$. For a partial converse, we recall from [15] the following purely algebraic result.

### Lemma 6.2

Let ${\mathfrak {Y}}\in \Gamma (M,(T^\prime M)^*)$. Then there exists a unique vector field $X\in \Gamma (M,TM)$ such that ${\mathfrak {Y}}$ is induced by *X* if and only if ${\mathfrak {Y}}(\tau )=\overline{{\mathfrak {Y}}(\tau )}$ for all characteristic forms $\tau$.

From now on, we shall not distinguish between *X* being a real vector field and a holomorphic vector field.

We recall the well-known identity, see e.g. [16],

$$\begin{aligned} {\mathcal {L}}_X\alpha (Y)=d\alpha (X,Y)+Y\alpha (X)=X\alpha (Y)-\alpha ([X,Y]), \end{aligned}$$

which holds for arbitrary complex vector fields *X*, *Y* and complex forms $\alpha$ on smooth manifolds. We conclude that accordingly the Lie derivative

$$\begin{aligned} {\mathcal {L}}_L\omega (\,.\,)=d\omega (L,\,.\,) \end{aligned}$$

of a holomorphic form $\omega$ with respect to a CR vector field *L* is again a holomorphic form. It is now possible to make the following definition. We shall say that a holomorphic vector field ${\mathfrak {Y}}\in \Gamma (M,(T^\prime M)^*)$ is CR iff

$$\begin{aligned} L\omega ({\mathfrak {Y}})=d\omega (L,{\mathfrak {Y}}) \end{aligned}$$

for every CR vector field *L* and holomorphic form $\omega$. In particular, a real vector field *X* is CR if and only if

$$\begin{aligned} \omega ([L,X])=0 \end{aligned}$$

for all CR vector fields *L* and holomorphic forms $\omega$. We recall from [15] the following fact.

### Proposition 6.3

If *X* is an infinitesimal CR automorphism on *M*, then *X* considered as a holomorphic vector field, i.e. $X\in {\mathcal {C}}^1(M,(T^\prime M)^*)$ is CR.

We are now able to generalise the notion of infinitesimal CR automorphism. To this end consider the space ${\mathcal {D}}^\prime (M,(T^\prime M)^*)$ of distributions with values in $(T^\prime M)^*$.

### Definition 6.4

An infinitesimal CR diffeomorphism of *M* with distributional coefficients is a generalised holomorphic vector field ${\mathfrak {Y}}\in {\mathcal {D}}^\prime (M,(T^\prime M)^*)$ that satisfies

6.1 $$\begin{aligned} L\omega ({\mathfrak {Y}})=({\mathcal {L}}_L\omega )({\mathfrak {Y}}) \end{aligned}$$

for every CR vector field *L* and holomorphic form $\omega$ and

6.2 $$\begin{aligned} {\mathfrak {Y}}(\tau )=\overline{{\mathfrak {Y}}(\tau )} \end{aligned}$$

for all characteristic forms $\tau$.

Note that (6.1) is in fact a CR equation for ${\mathfrak {Y}}$. If $U\subseteq M$ is an open subset of *M* then we say that ${\mathfrak {Y}}\in {\mathcal {D}}^\prime (M,(T^\prime M)^*)$ is an infinitesimal CR automorphism on *U* iff (6.1) and (6.2) hold for all local sections $L\in {\mathcal {E}}_{\mathcal {M}}(U,{\mathcal {V}}\vert _U)$ and $\theta \in {\mathcal {E}}_{\mathcal {M}}(U,T^0M\vert _U)$, respectively. Let the subset $U\subseteq M$ be small enough such that there is a local basis $L_1,\cdots , L_n$ of CR vector fields and also a local basis $\omega ^1,\cdots ,\omega ^N$ of the space of holomorphic forms. We recall that locally a distribution ${\mathfrak {Y}}\in {\mathcal {D}}^\prime (M,(T^\prime M)^*)$ is of the form

6.3 $$\begin{aligned} {\mathfrak {Y}}\vert _U=\sum _{j=1}^N X_j\omega ^j \end{aligned}$$

with $X_j\in {\mathcal {D}}^\prime (U)$. We introduce also the following operators on *U*

$$\begin{aligned} {\mathbf {L}}_j=L_j\cdot {{\mathbf {I}}}{{\mathbf {d}}}_N =\begin{pmatrix} L_j &{} &{}0\\ &{}\ddots \\ 0 &{} &{} L_j \end{pmatrix} \end{aligned}$$

and note that since $d\omega ^k (L_j,\,.\,)$ is again a holomorphic form we have

$$\begin{aligned} d\omega ^k(L_j,\,.\,)=\sum _{\ell =1}^N B_{k,\ell }^j \omega ^\ell \end{aligned}$$

with $B_{j,\ell }^k\in {\mathcal {E}}_{\mathcal {M}}(U)$. We observe that ${\mathfrak {Y}}$ is CR on *U* if and only if

$$\begin{aligned} L_j X_k=L_j\bigl (\omega ^k({\mathfrak {Y}})\bigr )=d\omega ^k\bigl (L_j,{\mathfrak {Y}})\bigr )=\sum _{\ell =1}^NB_{k,\ell }^j X_\ell \end{aligned}$$

for all $1\le j\le n$ and $0\le k\le N$. We set

$$\begin{aligned} B_j= \begin{pmatrix} B_{j,1}^1&{}\dots &{}B_{j,N}^1\\ \vdots &{} &{} \vdots \\ B_{j,1}^N &{}\dots &{}B_{j,N}^N \end{pmatrix}. \end{aligned}$$

Furthermore, using its local representation (6.3), we can identify ${\mathfrak {Y}}$ with the vector $X=(X_1,\dots ,X_N)$. Hence (6.1) turns into

$$\begin{aligned} {\mathbf {L}}_j X=B_j\cdot X \end{aligned}$$

or

$$\begin{aligned} P_j X=0, \end{aligned}$$

respectively, where

$$\begin{aligned} P_j= {\mathbf {L}}_j - B_j. \end{aligned}$$

In particular, we infer from above and Theorem 3.5 that

6.4 $$\begin{aligned} {{\,\mathrm{WF}\,}}_{\mathcal {M}}{\mathfrak {Y}}\subseteq T^0M. \end{aligned}$$

### Definition 6.5

Let $(M,{\mathcal {V}})$ be an ultradifferentiable abstract CR manifold of class $\{{\mathcal {M}}\}$, and ${\mathfrak {Y}}$ an infinitesimal CR diffeomorphism of *M* with distributional coefficients.

We say that ${\mathfrak {Y}}$ extends microlocally to a wedge with edge *M* iff there exists a set $\Gamma \subseteq T^0 M$ such that for each $p\in M$, the fibre $\Gamma _p \subseteq T^0_p M\setminus \{0\}$ is a closed, convex cone, and

$$\begin{aligned} {{\,\mathrm{WF}\,}}_{{\mathcal {M}}} (\omega ({\mathfrak {Y}})) \subseteq \Gamma \end{aligned}$$

for every holomorphic form $\omega \in {\mathcal {E}}_{{\mathcal {M}}}(M,T'M)$.

### Theorem 6.6

Let $(M,{\mathcal {V}})$ be an ultradifferentiable abstract CR structure of class $\{{\mathcal {M}}\}$, and ${\mathfrak {Y}}$ an infinitesimal CR diffeomorphism of *M* with distributional coefficients which extends microlocally to a wedge with edge *M*. Then, for any $\omega \in E$, the evaluation $\omega ({\mathfrak {Y}})$ is ultradifferentiable, and for any $\lambda \in {\mathcal {S}}$, the vector field $\lambda {\mathfrak {Y}}$ is also of class $\{{\mathcal {M}}\}$.

### Proof

Since the assertion is local we will work in a suitable small open set $U\subseteq M$ such that there are local bases $L_1,\cdots ,L_n$ of ${\mathcal {E}}_{\mathcal {M}}(U,{\mathcal {V}})$ and $\omega ^1,\cdots ,\omega ^N$ of ${\mathcal {E}}_{\mathcal {M}}(U,T^\prime M)$, respectively. We recall that we can represent ${\mathfrak {Y}}$ on *U* by (6.3) or by $X=(X_1,\cdots ,X_N)\in {\mathcal {D}}^\prime (U,{\mathbb {C}}^N)$. By assumption we know that there is a closed convex cone $\Gamma \subseteq T^0M\setminus \{0\}$ such that ${{\,\mathrm{WF}\,}}_{\mathcal {M}}X_j\subseteq \Gamma$ for each $j=1,\cdots ,N$. If we set $W^+=(\Gamma )^c\subseteq T^0M\setminus \{0\}$, then ${{\,\mathrm{WF}\,}}_{\mathcal {M}}X_j\cap W^+=\emptyset$ for all $j=1,\cdots ,N$. We may refer to this fact by saying that $X_j$ extends above. On the other hand, if we analogously put $W^-=(-\Gamma )^c\subseteq T^0M\setminus \{0\}$ then ${{\,\mathrm{WF}\,}}_{\mathcal {M}}{\bar{X}}_j\cap W^-=\emptyset$ by (3.6); we say that ${\bar{X}}_j$ extends below.

Furthermore, let $\{\theta ^1,\cdots , \theta ^d\}$ be a generating set of ${\mathcal {E}}_{\mathcal {M}}(U,T^0M)$ and recall (4.2), i.e.

$$\begin{aligned} {\mathcal {L}}^\alpha \theta ^\nu = \sum _{\ell =1}^N A^{\alpha , \nu }_\ell \omega ^\ell \end{aligned}$$

with $A^{\alpha ,\nu }_\ell \in {\mathcal {E}}_{\mathcal {M}}(U)$ for $\alpha \in {\mathbb {N}}^n_0$ and $\nu =1,\cdots , d$. In particular, (6.2), i.e. $\theta ({\mathfrak {Y}})=\overline{\theta ({\mathfrak {Y}})}$, turns into

$$\begin{aligned} \sum _{\ell =1}^N A^{0,\nu }_\ell X_\ell =\sum _{\ell =1}^N {\bar{A}}_\ell ^{0,\nu }{\bar{X}}_\ell \end{aligned}$$

and applying ${\mathcal {L}}^\alpha$ to (6.2) yields

$$\begin{aligned} \sum _{\ell =1}^N A^{\alpha ,\nu }_\ell X_\ell =\sum _{\ell =1}^N \sum _{|\beta |\le |\alpha |} C^{\beta ,\nu }_\ell L^\beta {\bar{X}}_\ell , \end{aligned}$$

where $C^{\beta ,\nu }_\ell \in {\mathcal {E}}_{\mathcal {M}}(U)$. Note that in both equations above the left-hand side extends above, while the right-hand side extends below.

Now choose any *N*-tuple ${\underline{\alpha }}=(\alpha ^1,\cdots ,\alpha ^N)\in {\mathbb {N}}_0^{Nn}$ of multi-indices with $|\alpha |\le k$ for all $j=1,\cdots ,N$ and $r=(r_1,\cdots ,r_N)\in \{1,\cdots ,d\}^N$. Then we have

$$\begin{aligned} \begin{pmatrix} A^{\alpha ^1,r_1}_1 &{} \dots &{} A^{\alpha ^1,r_1}_N \\ \vdots &{}\ddots &{} \vdots \\ A^{\alpha ^N,r_N}_1 &{} \dots &{} A^{\alpha ^N,r_N}_N \end{pmatrix} \begin{pmatrix} X_1 \\ \vdots \\ X_N \end{pmatrix} = \begin{pmatrix} \sum C^{\alpha ^1, \ell }_\beta L^\beta {\bar{X}}_\ell \\ \vdots \\ \sum C^{\alpha ^N, \ell }_\beta L^\beta {\bar{X}}_\ell \end{pmatrix}. \end{aligned}$$

If we multiply the equation with the classic adjoint of the matrix,

$$\begin{aligned} \begin{pmatrix} A^{\alpha ^1,r_1}_1 &{} \dots &{} A^{\alpha ^1,r_1}_N \\ \vdots &{}\ddots &{} \vdots \\ A^{\alpha ^N,r_N}_1 &{} \dots &{} A^{\alpha ^N,r_N}_N \end{pmatrix} \end{aligned}$$

then we obtain

$$\begin{aligned} D({\underline{\alpha }},r) X_j = \sum _{\begin{array}{c} |\beta |\le k \\ \ell =1,\dots , N \end{array}} D^{{\underline{\alpha }},r}_{\beta ,j } L^\beta {{\bar{X}}}_j \end{aligned}$$

for each $j=1,\cdots , N$ where the $D^{{\underline{\alpha }},r}_{\beta ,j }$ are ultradifferentiable functions on *U*. It follows that the right-hand side of this equation extends below, whereas the left-hand side obviously extends above. Hence ${{\,\mathrm{WF}\,}}_{\mathcal {M}}D({\underline{\alpha }},r)X=\emptyset$. We conclude that $\lambda X\in {\mathcal {E}}_{\mathcal {M}}(U)$ for any $\lambda \in {\mathcal {S}}^k(U)$ since ${\mathcal {S}}^k(U)$ is generated by the functions $D({\underline{\alpha }},r)$.    $\square$

The next statement is an obvious corollary of Theorem 6.6.

### Corollary 6.7

Let $(M,{\mathcal {V}})$ be finitely nondegenerate and *X* an infinitesimal CR diffeomorphism of *M* with distributional coefficients which extends microlocally to a wedge with edge *M*. Then *X* is ultradifferentiable of class $\{{\mathcal {M}}\}$.

However, the condition that *M* is actually finitely nondegenerate is far too restrictive. We shall say that $(M,{\mathcal {V}})$ is CR regular if for every $p\in M$ there exists a multiplier $\lambda \in {\mathcal {S}}$ of the form $\lambda (x)=x^\alpha {\tilde{\lambda }}(x)$ and ${\tilde{\lambda }}(p)\ne 0$ in some local coordinates *x* near *p*. Thence we can apply Proposition 2.12.

### Theorem 6.8

Let $(M,{\mathcal {V}})$ be an abstract CR structure, $p\in M$, and assume that *M* is CR regular near *p*. Then any locally integrable infinitesimal CR diffeomorphism *X* of *M* which extends microlocally to a wedge with edge *M* is of class $\{{\mathcal {M}}\}$ near *p*.

In general it might be difficult to determine if a certain CR manifold is CR regular. In the following, we want to present some instances of CR regular manifolds. But first we take a closer look at the Lie derivatives of characteristic forms.

Suppose that *M* is a CR manifold and near a point $p_0\in M$ there are local coordinates (*x*, *y*, *s*) of *M* such that the vector fields

6.5 $$\begin{aligned} L_j=\frac{\partial }{\partial {\bar{z}}_j}-\sum _{\tau =1}^d b^j_\tau \frac{\partial }{\partial s_\tau },\quad j=1,\cdots , n,\;z_j=x_j+y_j, \end{aligned}$$

where $b^j_\tau \in {\mathcal {E}}_{\mathcal {M}}$, are a local basis of CR vector fields near $p_0$. In this setting (which includes, for example, generic manifolds with the local coordinates from Proposition 4.1, see [2, § 1.6]), the characteristic bundle is spanned by the forms

$$\begin{aligned} \theta ^\tau = ds_\tau +\sum _{j=1}^n b^j_\tau \,d{\bar{z}}_j+\sum _{j=1}^n{\bar{b}}^j_\tau \,dz_j,\quad \tau =1,\cdots , d. \end{aligned}$$

Furthermore, the forms $\theta ^\tau$, $\tau =1,\cdots , d$ and $\omega ^j=dz_j$, $j=1,\cdots , n$, constitute a local basis of holomorphic forms on *M* near $p_0$. We also define the functions

$$\begin{aligned} \lambda ^{j,k}_\mu := L_k {\bar{b}}^j_\mu -{\bar{L}}_j b^k_\mu \end{aligned}$$

for $$j,k=1,\cdots , n$$ and $$\mu =1,\cdots ,d$$.

Consider a general holomorphic form

$$\begin{aligned} \eta =\sum _{\mu =1}^{d}\sigma _\mu \theta ^\mu +\sum _{j=1}^{n}\rho _j\omega ^j. \end{aligned}$$

The Lie derivative of $\eta$ with respect to the CR vector field $L_k$ is

6.6 $$\begin{aligned} {\mathcal {L}}_k\eta =d\eta (L_k,\,.\,)= \sum _{\mu =1}^d\Biggl (L_k\sigma _\mu -\sum _{\nu =1}^d\sigma _\nu \bigl (b^k_\nu \bigr )_{s_\mu }\Biggr )\theta ^\mu +\sum _{j=1}^n\Biggl (L_k\rho _j +\sum _{\mu =1}^d \sigma _\mu \lambda ^{j,k}_\mu \Biggr )\omega ^j.\nonumber \\ \end{aligned}$$

Let $(\alpha =(\alpha _1,\cdots ,\alpha _n)\in {\mathbb {N}}_0^n)$ be a multi-index of length $|\alpha |=m$. We introduce the finite sequence $m_j:=\sum _{\ell \le j}\alpha _{\ell }$, $j=1,\cdots ,n$, and set $m_0:=0$ and associate to $\alpha$ the function $p_\alpha :\,\{0,1,\cdots ,m\}\rightarrow \{0,1,\cdots ,n\}$ which is defined by

$$\begin{aligned} p_\alpha (\ell )=j \qquad \text {if }\; \ell \in (m_{j-1},m_j] \end{aligned}$$

for $\ell =1,\cdots ,m$ and $p_\alpha (0)=0$. We also associate the following sequences of multi-indices to $\alpha$

$$\begin{aligned} \alpha (\ell )&:=\sum _{q\le \ell } e_{p_\alpha (q)}&\ell&=0,1,\cdots ,m,\\ {\hat{\alpha }}(\ell )&:=\sum _{q>\ell } e_{p(q)}, \end{aligned}$$

where $e_j$ is the *j*-th standard unit vector in ${\mathbb {R}}^n$.

With this notation and (6.6), we can now state what the Lie derivative of the characteristic form $\theta ^\mu$ ($\mu =1,\cdots ,d$) is:

6.7 $$\begin{aligned} {\mathcal {L}}^\alpha \theta ^\mu =\sum _{\tau =1}^{d}T^{\alpha ,\mu }_{\tau }\theta ^\tau +\sum _{j=1}^n A^{\alpha ,\mu }_j\omega ^j. \end{aligned}$$

The functions $T^{\alpha ,\mu }_\tau$ and $A^{\alpha ,\mu }_j$ are defined iteratively by

6.8a $$\begin{aligned} T^{0,\mu }_\tau&=\delta _{\mu \tau },\nonumber \\ T^{\alpha ,\mu }_{\tau }&=L_{p_\alpha (1)}T^{{\hat{\alpha }}(1),\mu }_\tau -\sum _{\nu =1}^{d}\bigl (b^{p(1)}_\nu \bigr )_{s_\tau }T^{{\hat{\alpha }}(1),\mu }_\nu \end{aligned}$$

and

6.8b $$\begin{aligned} A^{\alpha ,\mu }_{j} =\sum _{k=1}^{m}\sum _{\nu =1}^{d}L^{\alpha (k-1)}\Bigl (T^{\alpha -\alpha (k),\mu }_\nu \lambda ^{j,p_\alpha (k)}_\nu \Bigr ). \end{aligned}$$

We are now able to give the first example of a CR regular submanifold of ${\mathbb {C}}^N$.

### Definition 6.9

We say that a real hypersurface $M\subseteq {\mathbb {C}}^N$ is weakly nondegenerate at $p_0$ iff there exist coordinates $(z,w)\in {\mathbb {C}}^n\times {\mathbb {C}}$ near $p_0$ and numbers $k,m\in {\mathbb {N}}$ such that $p_0=0$ in these coordinates and *M* is given near $p_0$ by an equation of the form

$$\begin{aligned} {{\,\mathrm{Im}\,}}w = ({{\,\mathrm{Re}\,}}w)^{m} \varphi (z,{{\bar{z}}}, {{\,\mathrm{Re}\,}}w), \end{aligned}$$

where

$$\begin{aligned} \frac{\partial ^{|\alpha | }\varphi }{\partial z^\alpha } (0,0,0) = \frac{\partial ^{|\alpha |}\varphi }{\partial {{\bar{z}}}^\alpha } (0,0,0) = 0, \quad |\alpha | \le k, \end{aligned}$$

and

$$\begin{aligned} {{\,\mathrm{span}\,}}_{\mathbb {C}}\{ \varphi _{z{{{\bar{z}}}}^\alpha } (0,0,0) :|\alpha |\le k \} = {\mathbb {C}}^n. \end{aligned}$$

If $k_0$ is the smallest *k* for which the preceding condition holds, we say that *M* is weakly $k_0$-nondegenerate at $p_0$.

### Proposition 6.10

Let $M\subseteq {\mathbb {C}}^N$ be an ultradifferentiable real hypersurface, $p_0\in M$, and assume that *M* is weakly $k_0$-nondegenerate at $p_0$. Then *M* is CR regular near $p_0$. In particular, any locally integrable infinitesimal CR diffeomorphism of *M* which extends microlocally to a wedge with edge *M* near $p_0$ is ultradifferentiable near $p_0$.

### Proof

In order to show that *M* is CR regular, we are going to construct a multiplier $\lambda \in {\mathcal {S}}$ of the form

$$\begin{aligned} \lambda (z,{\bar{z}},s)=s^\ell \psi (z,{\bar{z}},s) \end{aligned}$$

in suitable local coordinates and with $\psi \in {\mathcal {E}}_{\mathcal {M}}$ not vanishing at $s=0$ and $\ell \in {\mathbb {N}}$.

By assumption there are coordinates $(z,w)\in {\mathbb {C}}^n\times {\mathbb {C}}$ such that $p_0=0$ and *M* is given locally by

$$\begin{aligned} {{\,\mathrm{Im}\,}}w=({{\,\mathrm{Re}\,}}w)^m\varphi (z,{\bar{z}},{{\,\mathrm{Re}\,}}w) \end{aligned}$$

where $m\in {\mathbb {N}}$ and $\varphi$ is an ultradifferentiable real-valued function defined near 0 with the property that $\varphi _{z^\alpha }(0)=\varphi _{{\bar{z}}^\alpha }(0)=0$ for $|\alpha |\le k_0$ and

$$\begin{aligned} {{\,\mathrm{span}\,}}_{\mathbb {C}}\{ \varphi _{z{{{\bar{z}}}}^\alpha } (0,0,0) :0<|\alpha |\le k_0\} = {\mathbb {C}}^n. \end{aligned}$$

In these coordinates, a local basis of the CR vector fields on *M* is given by

$$\begin{aligned} L_j=\frac{\partial }{\partial {\bar{z}}_j}-b^j\frac{\partial }{\partial s},\qquad 1\le j\le n, \end{aligned}$$

with

$$\begin{aligned} b^j=i\frac{s^m\varphi _{{\bar{z}}_j}}{1+i(s^m\varphi )_s}, \end{aligned}$$

whereas the characteristic bundle is spanned near the origin by

$$\begin{aligned} \theta =ds+\sum _{j=1}^n b^j\,d{\bar{z}}_j +\sum _{j=1}^n b^j\,dz_j \end{aligned}$$

and $\theta$ together with the forms $\omega ^j=dz_j$ constitute a local basis of $T^\prime M$ near the origin.

We observe that for $1\le j,\ell \le n$

$$\begin{aligned} \lambda ^{j}_\ell&:=L_j{\bar{b}}^\ell -{\bar{L}}_\ell b^j\\&=s^m\Biggl (\frac{i\varphi _{{\bar{z}}_j z_\ell } (1+i(s^m\varphi )_s)+ \varphi _{z_\ell }(s^m\varphi _{{\bar{z}}_j})_s}{(1+i(s^m\varphi )_s)^2} +\frac{\varphi _{{\bar{z}}_j}\bigl ((s^m\varphi _{z_\ell })_s(1+i(s^m\varphi )_s) -is^m\varphi _{z_\ell }(s^m\varphi )_{ss}\bigr )}{(1+i(s^m\varphi )_s)^3}\\&\qquad \quad +\frac{i\varphi _{{\bar{z}}_j z_\ell } (1+i(s^m\varphi )_s) + \varphi _{{\bar{z}}_j}(s^m\varphi _{z_\ell })_s}{(1+i(s^m\varphi )_s)^2} -\frac{\varphi _{z_\ell }\bigl ((s^m\varphi _{{\bar{z}}_j})_s(1+i(s^m\varphi )_s) -s^m\varphi _{{\bar{z}}_j}(s^m\varphi )_{ss}\bigr )}{(1+i(s^m\varphi )_s)^3}\Biggr )\\&=s^m\chi ^j_\ell \end{aligned}$$

and $\chi ^j_\ell (0)=2i\varphi _{{\bar{z}}_jz_\ell }(0)$ by the assumptions on $\varphi$.

In this setting, (6.7) takes the form

$$\begin{aligned} {\mathcal {L}}^\alpha \theta =T^\alpha \theta +\sum _{j=1}^n A^\alpha _j\omega ^j \end{aligned}$$

and (6.8) implies that

$$\begin{aligned} T^\alpha&=L_{p(1)}T^{{\hat{\alpha }}(1)}-\bigl (b^{p(1)}\bigr )_s T^{{\hat{\alpha }}(1)}, \quad T^0=1,\\ A^\alpha _j&=\sum _{k=1}^{|\alpha |}=L^{\alpha (k-1)}\Bigl (T^{{\hat{\alpha }}(k)}\lambda ^j_{p(k)}\Bigr ). \end{aligned}$$

If we use two simple facts for smooth functions *f*, *g*, namely $(s^qf)_s=s^{q-1}f+s^qf_s$ for $q\in {\mathbb {N}}$, we see that $T^\beta =s^{m-1}G^\beta$ for $|\beta |\ge 1$. Hence, if $m\ge 2$ we have

$$\begin{aligned} A^{\alpha }_\ell (z,{\bar{z}},s)=s^m \frac{2i\varphi _{{\bar{z}}^\alpha z_\ell }(z,{\bar{z}},s)}{1+(s^m\varphi (z,{\bar{z}},s))_s^2} +s^{2m-1}R^\alpha _\ell (z,{\bar{z}},s)=s^m B^{\alpha }_\ell (z,{\bar{z}},s). \end{aligned}$$

On the other hand, we obtain for $m=1$ the following representation

$$\begin{aligned} A^{\alpha }_\ell (z,{\bar{z}},s)= & {} s\frac{2i\varphi _{{\bar{z}}^\alpha z_\ell }(z,{\bar{z}},s)}{1+(\varphi (z,{\bar{z}},s)+s\varphi _s(z,{\bar{z}},s))^2}\\&+sS^{\alpha }_\ell (z,{\bar{z}},s)+s^2R^{\alpha }_\ell (z,{\bar{z}},s)=sB^{\alpha }_\ell (z,{\bar{z}},s), \end{aligned}$$

where $S^{\alpha }_\ell$ is a sum of products of rational functions with respect to $\varphi$ and its derivatives. Each of these summands contains at least one factor of the form $\varphi _{{\bar{z}}^\beta }$ or $\varphi _{z^\beta }$ with $|\beta |\le |\alpha |\le k_0$ and therefore $S^{\alpha }_\ell (0)=0$.

By assumption, there have to be multi-indices $\alpha ^1,\dots ,\alpha ^n\ne 0$ of length shorter than $k_0$ such that

$$\begin{aligned} \{\varphi _{z{\bar{z}}^{\alpha ^1}}(0),\dots ,\varphi _{z{\bar{z}}^{\alpha ^n}}(0)\} \end{aligned}$$

is a basis for ${\mathbb {C}}^n$. Now we choose ${\underline{\alpha }}=(0,\alpha ^1,\dots ,\alpha ^n)$ and calculate according to (4.3) the multiplier $D({\underline{\alpha }})=D({\underline{\alpha }}, 1)$ (note that $d=1$):

$$\begin{aligned} D({\underline{\alpha }})&=\det \begin{pmatrix} 1 &{} 0 &{} \dots &{}0\\ A^{\alpha ^1}_\theta &{} A^{\alpha ^1}_1 &{} \dots &{} A^{\alpha ^1}_n\\ \vdots &{} \vdots &{} \ddots &{} \vdots \\ A^{\alpha ^n}_\theta &{} A^{\alpha ^n}_1 &{}\dots &{} A^{\alpha ^n}_n \end{pmatrix} =s^{n\cdot m}\det \begin{pmatrix} 1 &{} 0 &{} \dots &{}0\\ A^{\alpha ^1}_\theta &{} B^{\alpha ^1}_1 &{} \dots &{} B^{\alpha ^1}_n\\ \vdots &{} \vdots &{} \ddots &{} \vdots \\ A^{\alpha ^n}_\theta &{} B^{\alpha ^n}_1 &{}\dots &{} B^{\alpha ^n}_n \end{pmatrix}\\&=s^{n\cdot m}Q({\underline{\alpha }}),\\ \end{aligned}$$

where

$$\begin{aligned} Q({\underline{\alpha }})&=\det \begin{pmatrix} 1 &{} 0 &{} \dots &{}0\\ A^{\alpha ^1}_\theta &{} B^{\alpha ^1}_1 &{} \dots &{} B^{\alpha ^1}_n\\ \vdots &{} \vdots &{} \ddots &{} \vdots \\ A^{\alpha ^n}_\theta &{} B^{\alpha ^n}_1 &{}\dots &{} B^{\alpha ^n}_n \end{pmatrix} =\det \begin{pmatrix} B^{\alpha ^1}_1 &{} \dots &{} B^{\alpha ^1}_n\\ \vdots &{} \ddots &{} \vdots \\ B^{\alpha ^n}_1 &{}\dots &{} B^{\alpha ^n}_n \end{pmatrix},\\ \end{aligned}$$

hence

$$\begin{aligned} Q({\underline{\alpha }})(0)&=(2i)^n\det \begin{pmatrix} \varphi _{z{\bar{z}}^{\alpha ^1}}(0)\\ \vdots \\ \varphi _{z{\bar{z}}^{\alpha ^n}}(0) \end{pmatrix} \ne 0. \end{aligned}$$

We conclude that *M* is CR regular. $\square$

Obviously, a similar approach as in the hypersurface case above can be used to find manifolds of higher codimension that are CR regular.

### Definition 6.11

We say that a CR manifold $M\subseteq {\mathbb {C}}^{N}$ of codimension *d* is weakly nondegenerate at $p_0\in M$ (in the first codimension) iff there are local coordinates $(z,w)\in {\mathbb {C}}^{n+d}$ near $p_0$ such that *M* is given by the equations

$$\begin{aligned} {{\,\mathrm{Im}\,}}w_\mu = ({{\,\mathrm{Re}\,}}w)^{\gamma ^\mu } \varphi _\mu (z,{{\bar{z}}}, {{\,\mathrm{Re}\,}}w),\quad \mu =1,\cdots ,d, \end{aligned}$$

with $\gamma ^1<\gamma ^\nu$, $\nu =2,\cdots ,d$ and $|\gamma ^1|\ge 2$. Furthermore, the function $\varphi _1$ satisfies for some *k*

$$\begin{aligned} {{\,\mathrm{span}\,}}_{\mathbb {C}}\bigl \{\big (\varphi _1\big )_{z{\bar{z}}^\alpha }(0,0,0):\;|\alpha |\le k\bigr \}={\mathbb {C}}^n. \end{aligned}$$

If $k_0$ is the smallest integer *k* for which the above condition holds, we say that *M* is weakly $k_0$-nondegenerate at $p_0$.

### Proposition 6.12

Let $M\subseteq {\mathbb {C}}^{N}$ be a generic ultradifferentiable CR submanifold of codimension *d*, $p_0\in M$, and assume that *M* is weakly nondegenerate at $p_0$. Then any locally integrable infinitesimal CR diffeomorphism of *M* which extends microlocally to a wedge with edge *M* near $p_0$ is ultradifferentiable near $p_0$.

### Proof

Similar as before, we have to construct a multiplier $\lambda \in {\mathcal {S}}$ of the form $\lambda (z,{\bar{z}},s)=s^\beta \psi (z,{\bar{z}},s)$, where $\psi \in {\mathcal {E}}_{\mathcal {M}}$ and $\psi (0)\ne 0$. By assumption, there are coordinates $(z,w)\in {\mathbb {C}}^{n+d}$ near $p_0=0$ such that *M* is given by

$$\begin{aligned} {{\,\mathrm{Im}\,}}w_\mu = ({{\,\mathrm{Re}\,}}w)^{\gamma ^\mu } \varphi _\mu (z,{{\bar{z}}}, {{\,\mathrm{Re}\,}}w),\quad \mu =1,\cdots ,d. \end{aligned}$$

In particular, note that $\gamma ^1<\gamma ^\mu$ for $\mu =2,\cdots ,d$.

We use

$$\begin{aligned} L_j=\frac{\partial }{\partial {\bar{z}}_j}-\sum _{\mu =1}^d b^j_\mu \frac{\partial }{\partial s_\mu } \end{aligned}$$

as a local basis of the CR vector fields near the origin. The coefficients $b^j_\mu$ are of the form

$$\begin{aligned} b^j_\mu =i\big (\det ({{\,\mathrm{Id}\,}}_d +i\Phi )\big )^{-1} \cdot \det B^j_\mu \end{aligned}$$

where $\Phi$ denotes the Jacobi matrix of the map $(s^{\gamma ^\mu }\varphi _\mu )_\mu$ with respect to the variables $s=(s_1\cdots ,s_d)$ and

$$\begin{aligned} B^j_\mu =\begin{pmatrix} 1+i(s^{\gamma ^1}\varphi _1)_{s_1}&{}\dots &{} i(s^{\gamma ^1}\varphi _1)_{s_{\mu -1}} &{} s^{\gamma ^1}(\varphi _1)_{{\bar{z}}_j} &{} i(s^{\gamma ^1}\varphi _1)_{s_{\mu +1}} &{}\dots &{}i(s^{\gamma ^1}\varphi _1)_{s_d}\\ \vdots &{} &{}\vdots &{}\vdots &{}\vdots &{} &{}\vdots \\ i(s^{\gamma ^{\mu }}\varphi _{\mu })_{s_1}&{} \dots &{} i(s^{\gamma ^{\mu }}\varphi _{\mu })_{s_{\mu -1}} &{}s^{\gamma ^{\mu }}(\varphi _{\mu })_{{\bar{z}}_j} &{} i(s^{\gamma ^{\mu }}\varphi _{\mu })_{s_{\mu +1}} &{}\dots &{} i(s^{\gamma ^{\mu }}\varphi _{\mu })_{s_d}\\ \vdots &{}&{}\vdots &{}\vdots &{}\vdots &{}&{}\vdots \\ i(s^{\gamma ^d}\varphi _d)_{s_1}&{}\dots &{}i(s^{\gamma ^d}\varphi _d)_{s_{\mu -1}} &{}s^{\gamma ^d}(\varphi _d)_{{\bar{z}}_j} &{}i(s^{\gamma ^d}\varphi _d)_{s_{\mu +1}} &{}\dots &{} 1+i(s^{\gamma ^d}\varphi _d)_{s_d} \end{pmatrix}. \end{aligned}$$

Hence for all $j=1,\cdots n$ and $\mu =1,\cdots , d$ we have

6.9 $$\begin{aligned} b^j_\mu = is^{\gamma ^1}\big (\det ({{\,\mathrm{Id}\,}}_d +i\Phi )\big )^{-1}\det C^j_\mu \end{aligned}$$

with

$$\begin{aligned} C^j_\mu =\begin{pmatrix} 1+i(s^{\gamma ^1}\varphi _1)_{s_1}&{}\dots &{} i(s^{\gamma ^1}\varphi _1)_{s_{\mu -1}} &{} (\varphi _1)_{{\bar{z}}_j} &{} i(s^{\gamma ^1}\varphi _1)_{s_{\mu +1}} &{}\dots &{}i(s^{\gamma ^1}\varphi _1)_{s_d}\\ \vdots &{} &{}\vdots &{}\vdots &{}\vdots &{} &{}\vdots \\ i(s^{\gamma ^{\mu }}\varphi _{\mu })_{s_1}&{} \dots &{} i(s^{\gamma ^{\mu }}\varphi _{\mu })_{s_{\mu -1}} &{}s^{{\tilde{\gamma }}^{\mu }}(\varphi _{\mu })_{{\bar{z}}_j} &{} i(s^{\gamma ^{\mu }}\varphi _{\mu })_{s_{\mu +1}} &{}\dots &{} i(s^{\gamma ^{\mu }}\varphi _{\mu })_{s_d}\\ \vdots &{}&{}\vdots &{}\vdots &{}\vdots &{}&{}\vdots \\ i(s^{\gamma ^d}\varphi _d)_{s_1}&{}\dots &{}i(s^{\gamma ^d}\varphi _d)_{s_{\mu -1}} &{}s^{{\tilde{\gamma }}^d}(\varphi _d)_{{\bar{z}}_j} &{}i(s^{\gamma ^d}\varphi _d)_{s_{\mu +1}} &{}\dots &{} 1+i(s^{\gamma ^d}\varphi _d)_{s_d} \end{pmatrix} \end{aligned}$$

and ${\tilde{\gamma }}^\mu =\gamma ^\mu -\gamma ^1>0$. We observe that

6.10 $$ \begin{aligned} \det C^j_1\big \vert _{s=0}=(\varphi _1)_{{\bar{z}}_j}(z,{\bar{z}},0)\quad { \& } \quad \det C^j_\mu = 0, \;\,\mu =2,\cdots ,d, \end{aligned}$$

since $|\gamma ^\mu |\ge |\gamma ^1|\ge 2$. Furthermore, the forms

$$\begin{aligned} \theta ^\mu =ds_\mu +\sum _{j=1}^n b^j_\mu d{\bar{z}}_j +\sum _{j=1}^n{\bar{b}}^j_\mu dz_j,\quad \mu =1,\cdots ,d, \end{aligned}$$

span the characteristic bundle near 0 and $\theta ^\mu$, $\mu =1,\cdots ,d$ and $\omega ^j=dz_j$, $j=1,\cdots , n$, form a local basis of the holomorphic forms on *M*. From (6.7), we recall for $\alpha \in {\mathbb {N}}_0^n$ and $\mu =1,\cdots ,d$ that

$$\begin{aligned} {\mathcal {L}}^\alpha \theta ^\mu =\sum _{\tau =1}^{d}T^{\alpha ,\mu }_{\tau }\theta ^\tau +\sum _{j=1}^n A^{\alpha ,\mu }_j\omega ^j \end{aligned}$$

and from (6.8),

$$\begin{aligned} T^{0,\mu }_\tau&=\delta _{\mu \tau }\\ T^{\alpha ,\mu }_{\tau }&=L_{p_\alpha (1)}T^{{\hat{\alpha }}(1),\mu }_\tau -\sum _{\nu =1}^{d}\bigl (b^{p(1)}_\nu \bigr )_{s_\tau }T^{{\hat{\alpha }}(1),\mu }_\nu \\ A^{\alpha ,\mu }_{j}&=\sum _{k=1}^{|\alpha |} \sum _{\nu =1}^{d}L^{\alpha (k-1)}\Bigl (T^{\alpha -\alpha (k),\mu }_\nu \lambda ^{j,p_\alpha (k)}_\nu \Bigr ). \end{aligned}$$

We recall that

$$\begin{aligned} \lambda ^{j,k}_\nu =L_k{\bar{b}}^j_\nu -{\bar{L}}_jb^k_\nu =\big ({\bar{b}}^j_\nu \big )_{{\bar{z}}_k}-\sum _{\mu =1}^d b^k_\mu \big ({\bar{b}}^j_\nu \big )_{s_\mu } -\big (b^k_\nu \big )_{z_j}+\sum _{\mu =1}{\bar{b}}^j_\mu \big (b^k_\nu \big )_{s_\mu } \end{aligned}$$

and note that (6.9) and (6.10) imply thatwhere

$$\begin{aligned} R_1^{j,k}\Big \vert _{s=0}&=\big (\varphi _1\big )_{{\bar{z}}_kz_j}\Big \vert _{s=0}\\ R_\nu ^{j,k}\Big \vert _{s=0}&=0&\nu&=1,\cdots ,d. \end{aligned}$$

It is easy to see that also $T^{\alpha ,\mu }_\tau \big \vert _{s=0} =0$ for $\alpha \ne 0$. We conclude that for all $\alpha \ne 0$, and $j=1,\cdots ,n$where

$$\begin{aligned} {\tilde{A}}^{\alpha ,1}_j\Big \vert _{s=0}&=\big (\varphi _1\big )_{{\bar{z}}^\alpha z_j}\Big \vert _{s=0},\\ {\tilde{A}}^{\alpha ,\mu }_j\Big \vert _{s=0}&=0&\mu&=2,\cdots , d. \end{aligned}$$

By assumption there are multi-indices $\alpha ^1,\cdots ,\alpha ^n\in {\mathbb {N}}_0^n$ of length at most $k_0$ such that the vectors

$$\begin{aligned} \bigl (\varphi _1\bigr )_{z{\bar{z}}^{\alpha ^j}}(0),\qquad j=1,\cdots ,n, \end{aligned}$$

form a basis of ${\mathbb {C}}^n$.

We compute the multiplier $D({\overline{\alpha }},r)$ for ${\underline{\alpha }}=(0,\cdots ,0,\alpha ^1,\cdots ,\alpha ^n)$ and $r=(1,2,\cdots ,d,1,\cdots ,1)$. By (4.3) we have

$$\begin{aligned} D({\underline{\alpha }},r)&=\det \begin{pmatrix} 1&{}\dots &{}0&{}0&{}\dots &{}0\\ \vdots &{} &{}\vdots &{}\vdots &{}&{}\vdots \\ 0&{}\dots &{}1&{}0&{}\dots &{}0\\ T^{\alpha ^1,1}_1 &{}\dots &{}T^{\alpha ^1,1}_d&{} A^{\alpha ^1,1}_1&{}\dots &{}A^{\alpha ^1,1}_n\\ \vdots &{}&{}\vdots &{}\vdots &{}&{}\vdots \\ T^{\alpha ^n,1}_1 &{}\dots &{}T^{\alpha ^n,1}_d&{}A^{\alpha ^n,1}_1&{}\dots &{}A^{\alpha ^n,1}_n \end{pmatrix}\\&=\det \begin{pmatrix} A^{\alpha ^1,1}_1&{}\dots &{}A^{\alpha ^1,1}_n\\ \vdots &{}&{}\vdots \\ A^{\alpha ^n,1}_1&{}\dots &{}A^{\alpha ^n,1}_n \end{pmatrix} =\det \begin{pmatrix} 2is^{\gamma ^1}{\tilde{A}}^{\alpha ^1,1}_1&{}\dots &{}2is^{\gamma ^1}{\tilde{A}}_n^{\alpha ^1,1}\\ \vdots &{}&{}\vdots \\ 2is^{\gamma ^1}{\tilde{A}}_1^{\alpha ^n,1} &{}\dots &{}2is^{\gamma ^1}{\tilde{A}}_n^{\alpha ^n,1} \end{pmatrix}\\&=(2i)^n s^{n\gamma ^1}\det \begin{pmatrix} {\tilde{A}}^{\alpha ^1,1}_1 &{}\dots &{}{\tilde{A}}^{\alpha ^1,1}_n\\ \vdots &{}&{}\vdots \\ {\tilde{A}}^{\alpha ^n,1}_1 &{}\dots &{}{\tilde{A}}^{\alpha ^n,1}_n \end{pmatrix} =(2i)^n s^{n\gamma ^1}\Lambda ({\underline{\alpha }},r). \end{aligned}$$

We conclude

$$\begin{aligned} \Lambda ({\underline{\alpha }},r)(0)=\det \begin{pmatrix} \big (\varphi _1\big )_{z{\bar{z}}^{\alpha ^1}}(0)\\ \vdots \\ \big (\varphi _1\big )_{z{\bar{z}}^{\alpha ^n}}(0) \end{pmatrix}\ne 0. \end{aligned}$$

$$\square $$

In the preceding results, we required the involved manifolds to have a special form in order to simplify the necessary calculations, but of course there are many more CR regular manifolds. The next example gives a CR manifold that is not weakly nondegenerate at 0 in the sense of Definition 6.11 but is still CR regular.

### Example 6.13

Let $M\subseteq {\mathbb {C}}^3$ be the CR manifold given by

$$\begin{aligned} {{\,\mathrm{Im}\,}}w_1&= {{\,\mathrm{Re}\,}}w_1\,|z|^2,\\ {{\,\mathrm{Im}\,}}w_2&={{\,\mathrm{Re}\,}}w_2\,|z|^2. \end{aligned}$$

The CR bundle ${\mathcal {V}}$ of *M* is spanned by

$$\begin{aligned} L=\frac{\partial }{\partial {\bar{z}}}-i\frac{s_1z}{1+i|z|^2}\frac{\partial }{\partial s_1}-i\frac{s_2 z}{1+i|z|^2}\frac{\partial }{\partial s_2}. \end{aligned}$$

Thus a basis of the characteristic form is given by

$$\begin{aligned} \theta ^1&=ds_1+ i\frac{s_1z}{1+i|z|^2}d{\bar{z}}-i\frac{s_1{\bar{z}}}{1-i|z|^2}dz,\\ \theta ^2&=ds_2+ i\frac{s_2z}{1+i|z|^2}d{\bar{z}}-i\frac{s_2{\bar{z}}}{1-i|z|^2}dz. \end{aligned}$$

We know that $\theta ^1$, $\theta ^2$ and $\omega =dz$ form a basis of $T^\prime M$. If $\alpha =e_1$ we recall from (6.7) that

$$\begin{aligned} {\mathcal {L}}^\alpha \theta ^1=T^{\alpha ,1}_{1}\theta ^1 +T^{\alpha ,1}_{2}\theta ^2 + A^{\alpha ,1}\omega . \end{aligned}$$

Using (6.8), we observe that

$$\begin{aligned} T^{\alpha ,1}_1&=-i\frac{z}{1+i|z|^2}\\ T^{\alpha ,1}_2&=0\\ A^{\alpha ,1}&=-2is_1\frac{1-|z|^4}{\bigl (1+|z|^4\bigr )^2}. \end{aligned}$$

Hence, if we set ${\underline{\alpha }}=(0,0,\alpha )$ and $r=(1,2,1)$ then the multiplier $D({\underline{\alpha }},r)$ of *M* given by (4.3) is

$$\begin{aligned} D({\underline{\alpha }},r)=\det \begin{pmatrix} 1 &{} 0&{} 0\\ 0&{} 1&{} 0\\ -i\tfrac{z}{1+i|z|^2}&{}0 &{}-2is_1\tfrac{1-|z|^4}{(1+|z|^4)^2} \end{pmatrix} =-2is_1\frac{1-|z|^4}{\bigl (1+|z|^4\bigr )^2} \end{aligned}$$

and thus *M* is CR regular.

We could now give an ultradifferentiable version of the example given in section 7 of [15] in order to show that in the previous statements the requirement on the infinitesimal automorphisms to be locally integrable is essential for the assertions to hold. However, to do this it would be enough to replace everywhere in section 7 of [15] the word *smooth* with the term *ultradifferentiable of class*$\{{\mathcal {M}}\}$.

Instead we take a closer look into the case of quasianalytic manifolds. We begin with recalling the following definition from [2, § 11.7]. Let $M\subseteq {\mathbb {C}}^N$ be a CR submanifold with defining functions $\rho =(\rho _1,\cdots ,\rho _d)$ near $p_0\in M$. A formal holomorphic vector field at $p_0$ is a vector field of the form

$$\begin{aligned} X=\sum _{j=1}^N a_j(Z)\frac{\partial }{\partial Z_j} \end{aligned}$$

with the coefficients $a_j$ being formal power series in $Z-p_0$ with complex coefficients. The formal vector field *X* is said to be tangent to *M* at $p_0$ iff there exists a $d\times d$ matrix $c(Z,{\bar{Z}})$ consisting of formal power series in the variables $Z-p_0$ and ${\bar{Z}}-{\bar{p}}_0$ such that

$$\begin{aligned} X\rho (Z,{\bar{Z}})\sim c(Z,{\bar{Z}})\rho (Z,{\bar{Z}}), \end{aligned}$$

where $\sim$ denotes equality as formal power series in $Z-p_0$ and ${\bar{Z}}-{\bar{p}}_0$. Note that the existence of nontrivial holomorphic vector fields at $p_0$ tangent to *M* does not depend on the choice of holomorphic coordinates and defining equations near $p_0$.

### Definition 6.14

A generic submanifold $M\subseteq {\mathbb {C}}^N$ is formally holomorphically nondegenerate at $p_0\in M$ iff there is no nontrivial formal holomorphic vector field at $p_0$ that is tangent to *M*.

### Remark 6.15

If *M* is formally holomorphically nondegenerate at $p_0$ then *M* is formally holomorphically nondegenerate at every point of some neighbourhood *U* of $p_0$. Furthermore, if *M* is formally holomorphically nondegenerate on an open set $U\subseteq M$ then *M* is finitely nondegenerate on an open and dense subset $V\subseteq U$, c.f. [2, Theorem 11.7.5].

### Theorem 6.16

Let ${\mathcal {M}}$ be a quasianalytic normal weight sequence and $M\subseteq {\mathbb {C}}^N$ a generic submanifold of class $\{{\mathcal {M}}\}$ that is formally holomorphically nondegenerate. Every smooth CR diffeomorphism ${\mathfrak {Y}}$ that extends microlocally to a wedge with edge *M* is ultradifferentiable of class $\{{\mathcal {M}}\}$.

### Proof

As usual, we argue locally near a point $p_0$. After a choice of local bases of CR vector fields and holomorphic forms and selecting a generating set for the characteristic forms, we can use the representation (6.3) near $p_0$. By Theorem 6.6, we know that for any multiplier $\lambda$ the product $\Lambda _j =\lambda \cdot X_j$ is ultradifferentiable for $j=1,\cdots , N$. Since $X_j$ is smooth by assumption, we have that the equality holds also for the formal power series at $p_0$ of $\Lambda _j$, $\lambda$ and $X_j$. Since *M* is formally holomorphically nondegenerate at $p_0$, there has to be a multiplier $\lambda \in {\mathcal {S}}$ with nontrivial formal power series at $p_0$. Indeed, if the power series of $\lambda$ at $p_0$ equals 0 then $\lambda$ itself has to vanish in a neighbourhood of $p_0$ by the quasianalyticity of ${\mathcal {M}}$. On the other hand, in every neighbourhood of $p_0$ there is a point *q* at which *M* is finitely nondegenerate by [2, Theorem 11.7.5]. Hence by Remark 4.5, there has to be a nontrivial multiplier $\lambda ^\prime$ defined on some neighbourhood *U* of $p_0$. We conclude that the formal power series of $\Lambda _j^\prime =\lambda ^\prime X_j$ at $p_0$ is divisible by the Taylor series of $\lambda ^\prime$ at $p_0$. Hence the main result in [29] gives that $X_j$ is ultradifferentiable of class $\{{\mathcal {M}}\}$ near $p_0$. $\square$
